# Predictors of COVID-19 epidemics in countries of the World Health Organization African Region

**DOI:** 10.1038/s41591-021-01491-7

**Published:** 2021-09-03

**Authors:** Feifei Zhang, Humphrey Karamagi, Ngoy Nsenga, Miriam Nanyunja, Miriam Karinja, Seth Amanfo, Margo Chase-Topping, Giles Calder-Gerver, Miles McGibbon, Alexandra Huber, Tara Wagner-Gamble, Chuan-Guo Guo, Samuel Haynes, Alistair Morrison, Miranda Ferguson, Gordon A. Awandare, Francisca Mutapi, Zabulon Yoti, Joseph Cabore, Matshidiso R. Moeti, Mark E. J. Woolhouse

**Affiliations:** 1 Usher Institute, University of Edinburgh, Edinburgh, United Kingdom; 2 WHO Regional Office for Africa, Brazzaville, Republic of Congo; 3 University of Nairobi Institute of Tropical and Infectious Diseases, Nairobi, Kenya; 4 School of Biological Sciences, University of Edinburgh, Edinburgh, United Kingdom; 5 Roslin Institute and Royal (Dick) School of Veterinary Studies, University of Edinburgh, Edinburgh, United Kingdom; 6 Department of Medicine, Li Ka Shing Faculty of Medicine, University of Hong Kong, Hong Kong, China; 7 West African Centre for Cell Biology of Infectious Pathogens, University of Ghana, Accra, Ghana

**Keywords:** Risk factors, Epidemiology, Health policy

## Abstract

Countries of the World Health Organization (WHO) African Region have experienced a wide range of coronavirus disease 2019 (COVID-19) epidemics. This study aimed to identify predictors of the timing of the first COVID-19 case and the per capita mortality in WHO African Region countries during the first and second pandemic waves and to test for associations with the preparedness of health systems and government pandemic responses. Using a region-wide, country-based observational study, we found that the first case was detected earlier in countries with more urban populations, higher international connectivity and greater COVID-19 test capacity but later in island nations. Predictors of a high first wave per capita mortality rate included a more urban population, higher pre-pandemic international connectivity and a higher prevalence of HIV. Countries rated as better prepared and having more resilient health systems were worst affected by the disease, the imposition of restrictions or both, making any benefit of more stringent countermeasures difficult to detect. Predictors for the second wave were similar to the first. Second wave per capita mortality could be predicted from that of the first wave. The COVID-19 pandemic highlights unanticipated vulnerabilities to infectious disease in Africa that should be taken into account in future pandemic preparedness planning.

## Abstract (web summary)

A new study from the WHO African Region identifies features of countries that predict timing of the first case and the per capita mortality rate for the first and second waves of the COVID-19 epidemics.

## Main

COVID-19, caused by severe acute respiratory syndrome coronavirus 2 (SARS-CoV-2), presents a continuing threat to both global health and the global economy. By early March 2021, more than 119 million cases worldwide had been reported, with more than 2.6 million deaths^1^. Despite the implementation of unprecedented public health interventions, including social distancing, contact tracing and large-scale lockdowns of the population^2^, the burden of the disease has continued to rise but with substantial variation among countries and regions and with countries in many regions around the world experiencing multiple waves^1^. As of 14 March 2021, the WHO African Region had experienced two waves of infection and had reported a total of over 2.9 million cases of infection and more than 74,000 deaths^1,3^. A third wave is currently in progress.

Gaining an understanding of variation in the progression of the pandemic in different countries will aid the response to future pandemics. Current evidence from high- and middle-income countries suggests that demographics (for example, percentage of the population aged 65 years or older), comorbidities, healthcare resources and stringency of response are important risk factors for COVID-19-related infections^2,4–6^. It was suggested that Africa would be more susceptible to SARS-CoV-2-related cases and deaths given the higher prevalence of pre-existing conditions, including tuberculosis, malaria, AIDS, diabetes, undernourishment and other communicable and non-communicable comorbidities, as well as lower accessibility to healthcare^7,8^. Recent work suggests that spatial connectivity might also have an important influence on the course of the pandemic in Africa^9^. Using the data for COVID-19 cases and deaths from the WHO COVID-19 Dashboard, this study aimed to identify predictors of the timing of the first case and the per capita mortality rate in the first and second COVID-19 pandemic waves in the WHO African Region and to test for any effect of intervention measures on COVID-19-related deaths. We included, as predictors, existing indices of epidemic preparedness—COVID-19 readiness status and the more generic infectious diseases resilience index (Supplementary Table 1)—to test the expectation that countries rated as better prepared would suffer less severe outcomes. The main findings and limitations of the study are summarized in Table 1.

**Table 1:** Policy summary

Category	Description
Background	The direct and indirect effects of the COVID-19 pandemic have been highly heterogeneous across Africa. It is important to establish whether this variation is primarily driven by differences in intrinsic socio-ecological characteristics, responses to the pandemic or an artifact of differences in reporting.
Main findings and limitations	This observational study confirmed that early onsets of national COVID-19 epidemics were partly driven by international connectivity, whereas high urbanization, international connectivity and HIV/AIDS prevalence predicted high first wave mortality rate, which, in turn, was a predictor of high second wave mortality rate. Levels of preparedness and resilience, expected to reflect a causal relationship with effective pandemic management, instead had the opposite relationship. Our analysis corrected for estimated levels of under-reporting of COVID-19 deaths. However, varied levels of data availability and quality of reporting still remain a concern, particularly the mortality data gaps in some countries excluded from this analysis.
Policy implications	The observation that the COVID-19 pandemic has had a greater effect on WHO African Region countries perceived to be less vulnerable to infectious disease outbreaks challenges current definitions of ‘preparedness’ and ‘resilience’. More urbanized countries with stronger travel links and with more advanced healthcare systems were more vulnerable to COVID-19 mortality, contrary to expectations. This could be due to differences in access to healthcare, mismatches between investments in strong health systems vis-à-vis COVID-19 response needs and/or the syndemic nature of COVID-19 providing unique challenges. The possible association with the prevalence of HIV/AIDS requires further exploration, as multiple co-factors, such as poverty, other health conditions, different socio-economic status and other variables, could be correlated with this—although the finding is consistent with a broader pattern of a range of comorbidities having a significant effect on COVID-19 mortality rates. Although our results were found to be robust to variation in testing effort, there is evidence of under-reporting and a clear need for improved surveillance and death certification systems. The finding of no evidence that a more stringent policy response to the first wave reduced the size of the second wave in countries that experienced it is consistent with the risk factors that we identify as being difficult or impossible to mitigate against. The current and future introduction of variants of COVID-19 will either accentuate or dampen these effects depending on their relative infectiousness. Future emphasis should focus on a more comprehensive perspective of preparedness, mitigation and resilience.

## Results

### COVID-19 epidemics in countries of the WHO African Region

**Fig. 1:**
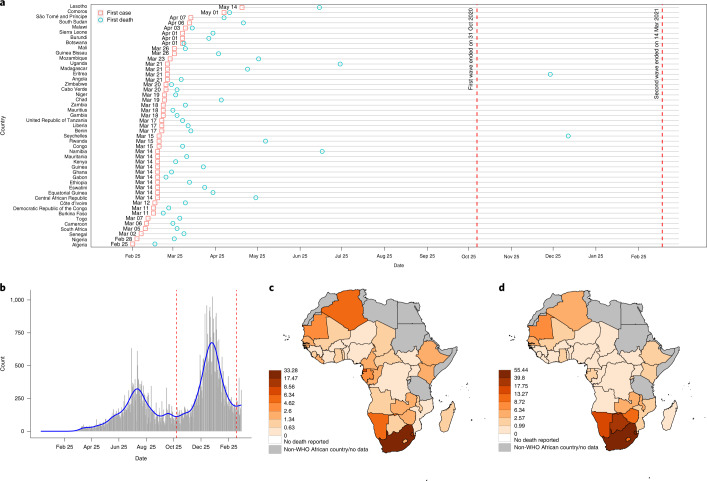
COVID-19 pandemic in the WHO African Region. **a**, Timeline of the first case and first death. **b**, Pandemic curve for daily new deaths. Map of per capita mortality rates in the first wave (**c**) and in the second wave (**d**). Tanzania, Burundi, Eritrea and Seychelles were excluded (Methods) and are shown in gray in **c** and **d**.

On 25 February 2020, Algeria was the first country in the WHO African Region to report COVID-19 cases (Fig. 1a). Thirty-one countries reported their first cases in the 2 weeks from 12 March to 26 March 2020. Lesotho was the last of the 47 countries to report its first case, on 14 May 2020. There was no apparent relationship between the timing of the first COVID-19 case and the first death (Fig. 1a).

**Fig. 2:**
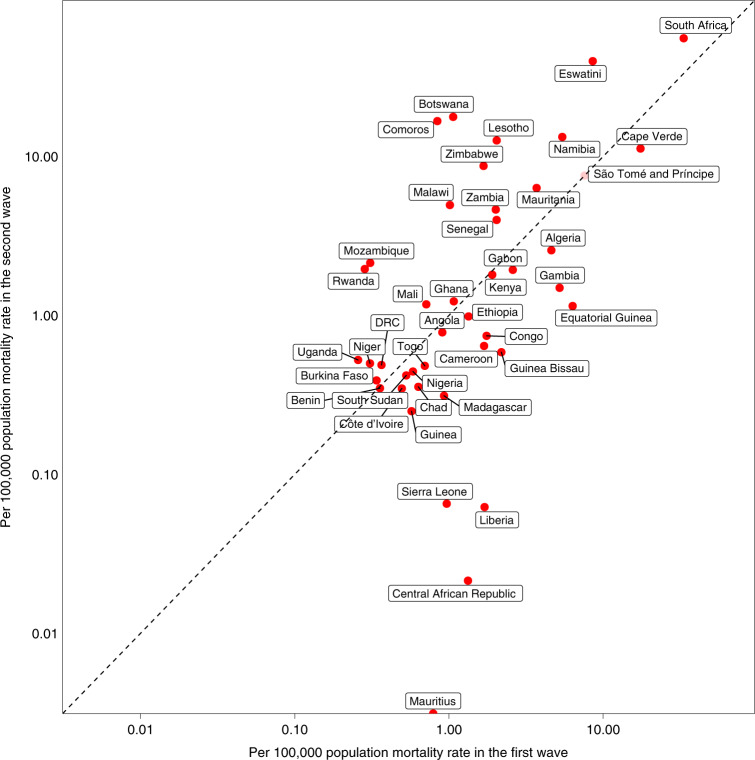
Scatter plot of per capita mortality in the first and second waves. Axes on log_10_ scale with points falling on the axes denoting zero deaths. The dashed line indicates identical levels of mortality rates in two waves. Tanzania, Burundi, Eritrea and Seychelles were not shown due to incomplete data/being outliers. Note that São Tomé and Principe was not included in mortality rate analyses due to missing predictor data. DRC, Democratic Republic of the Congo.

The 47 Member States reported a total of 29,635 COVID-19 deaths in the first wave and 44,850 deaths in the second wave. However, Tanzania discontinued reporting of COVID-19-related deaths from 8 May 2020, and Burundi, Eritrea and Seychelles were outliers (0.009, 0 and 0 per 100,000 population first wave mortality rates, respectively). São Tomé and Príncipe, as well as Seychelles, had missing data on the prevalence of HIV. These five countries were, therefore, excluded from the mortality rate analyses, giving a sample size of 42. Daily new deaths in the whole WHO African Region peaked on 5 August 2020 in the first wave and on 18 January 2021 in the second wave (Fig. 1b), lagging 16 and 7 d behind the peak of daily new cases in the first and second waves, respectively. The WHO African Region as a whole experienced a higher second wave peak than the first wave: 323 deaths (on 5 August 2020) and 675 (on 18 January 2021), respectively. In the first wave, the highest mortality per 100,000 population was reported from South Africa (33.3), followed by Cape Verde (17.5) and Eswatini (8.6) (Fig. 1c). In the second wave, the highest mortality per 100,000 population was also reported from South Africa (55.4), followed by Eswatini (39.8) and Botswana (17.7) (Fig. 1d). Twenty countries had higher or similar mortality rates in the second wave than in the first wave, whereas 23 countries had lower mortality rates in the second wave than in the first wave (Fig. 2).

### Predictors of the timing of the first case

**Fig. 3:**
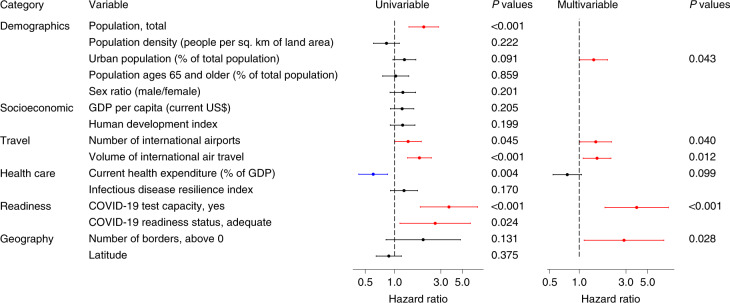
HRs and 95% CIs for predictors of timing of the first case in univariable and multivariable Cox regression model. *n* = 47 countries. Error bars are shown. Statistically significant risk factors are in red; protective factors are in blue. Exact two-sided *P* values for the Wald test are shown for each predictor, and two-sided *P* values < 0.05 were considered statistically significant.

**Extended Data Fig. 1:**
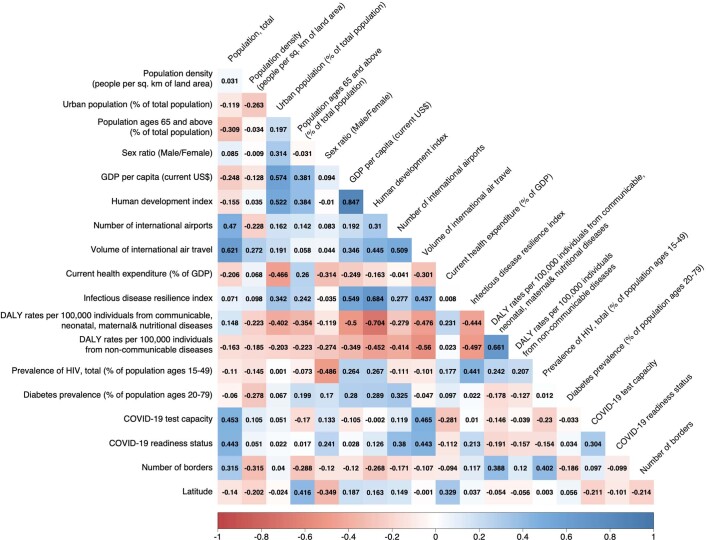
Correlation matrix for predictors in the first wave. Positive correlations are displayed in blue and negative correlations in red colour. *n* = 47 countries. Spearman’s rank correlation test was used. Colour intensity is proportional to the correlation coefficients.

We included 47 countries and 15 predictors (Supplementary Fig. 1a–k,p–s) in the Cox regression model for timing of the first case. Spearman’s correlation identified five pairs of predictors with correlation coefficients greater than 0.6 (Extended Data Fig. 1). The univariable Cox regression model identified total population size, number of international airports, volume of international air travel, COVID-19 test capacity and COVID-19 readiness status as risk factors for earlier detection of the first case and current health expenditure (percent of GDP) as protective factors (Fig. 3 and Supplementary Table 2). In the multivariable model, the percentage of urban population (hazard ratio (HR) = 1.40, 95% confidence interval (CI) 1.01–1.95), number of international airports (HR = 1.48, 95% CI 1.02–2.14), volume of international air travel (HR = 1.52, 95% CI 1.10–2.11), COVID-19 test capacity (HR = 3.86, 95% CI 1.83–8.15) and number of borders (HR = 2.87, 95% CI 1.12–7.32) were identified as risk factors for earlier detection of the first case (Fig. 3 and Supplementary Table 2).

### Predictors of per capita mortality during the first wave

**Fig. 4:**
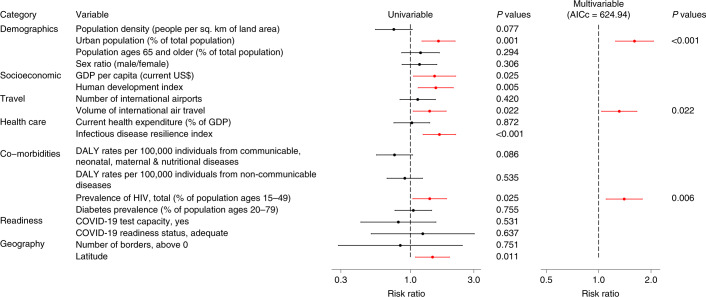
RRs and 95% CIs of predictors of per capita mortality in the first wave in univariable and multivariable Poisson GLMM. *n* = 42 countries. Error bars are shown. Statistically significant risk factors are in red. Exact two-sided *P* values for the Wald test are shown for each predictor, and two-sided *P* values < 0.05 were considered statistically significant.

We included 42 countries and 18 predictors (Supplementary Fig. 1b–s) in the generalized linear mixed models (GLMMs) for per capita mortality in the first wave. In the univariable analyses, the percentage of urban population, GDP per capita, human development index, volume of international air travel, infectious disease resilience index, prevalence of HIV and latitude were risk factors (Fig. 4 and Supplementary Table 3). The correlation between the time to first case and per capita mortality was not significant (*P* = 0.22). In the multivariable GLMM, the percentage of urban population (risk ratio (RR) = 1.61, 95% CI 1.25–2.06), volume of international air travel (RR = 1.31, 95% CI 1.04–1.66) and prevalence of HIV (RR = 1.40, 95% CI 1.10–1.78) were risk factors for mortality rate in the first wave (Fig. 4 and Supplementary Table 3). Percentage of urban population was included in all models within +2-corrected Akaike information criterion (AICc) scores (Methods); volume of international air travel and HIV prevalence were included in most but not all.

**Extended Data Fig. 2:**
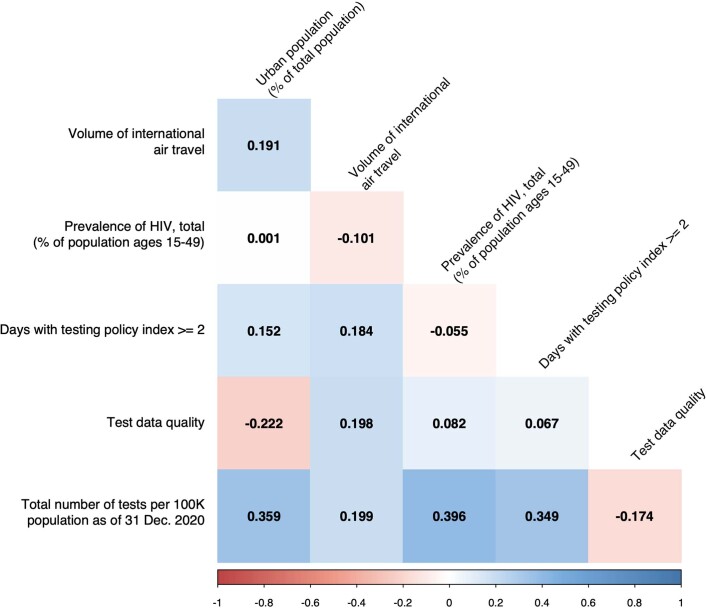
Correlation matrix for significant predictors in multivariable model for per capita mortality in the first wave and three test variables. Positive correlations are displayed in blue and negative correlations in red colour. *n* = 42 countries. Spearman’s rank correlation test was used. Colour intensity is proportional to the correlation coefficients.

**Extended Data Fig. 3:**
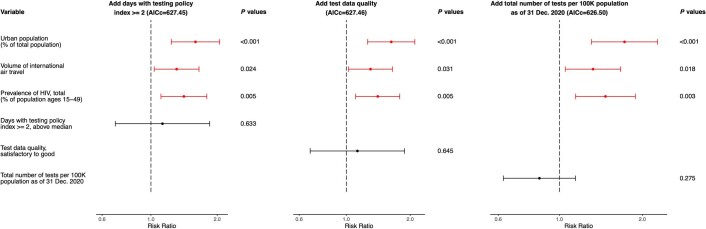
Risk ratios and 95% confidence intervals of three test variables for per capita mortality in the first wave in multivariable Poisson generalized linear mixed model. *n* = 42 countries. Error bars are shown. Statistically significant risk factors are in red. Exact two-sided *P* values for the Wald test are shown for each predictor, and two-sided *P* values < 0.05 were considered statistically significant.

None of the predictors in the best multivariable model was correlated with any of the COVID-19 testing variables (correlation coefficients < 0.6) (Extended Data Fig. 2). We then re-ran the best multivariable GLMMs with each additional testing variable (Supplementary Fig. 1u,w–x). No test variable was associated with the per capita mortality rate and reduced the AICc, and there were no changes in the RRs estimated by the best multivariable model (Extended Data Fig. 3 and Supplementary Table 3).

There was a good consistency between the stringency index and percent change of residential mobility as indicated by the Google mobility data. After controlling for temporal and random effects, the stringency index was non-linearly associated with the residential mobility (*P* < 0.0001), with an effective degree of freedom of 8.66. The *R*^2^ of the model is 0.77, and the explained deviance is 77.5%.

**Extended Data Fig. 4:**
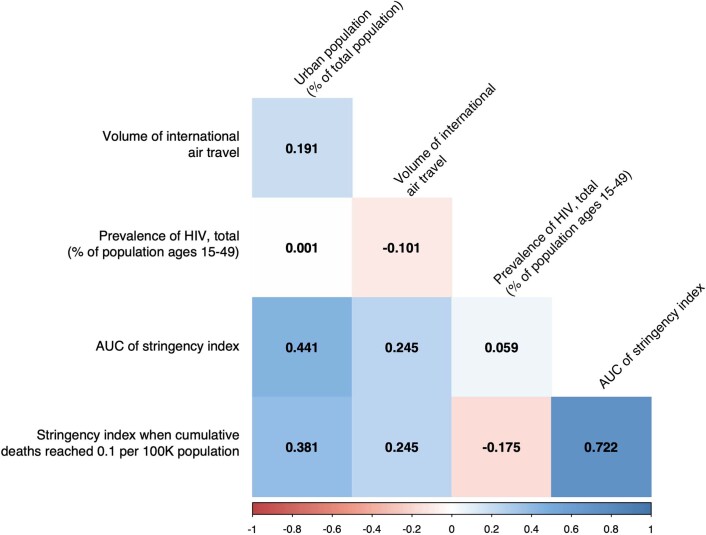
Correlation matrix for significant predictors in multivariable model for per capita mortality in the first wave and two stringency indices. Positive correlations are displayed in blue and negative correlations in red colour. *n* = 42 countries. Spearman’s rank correlation test was used. Colour intensity is proportional to the correlation coefficients.

**Extended Data Fig. 5:**
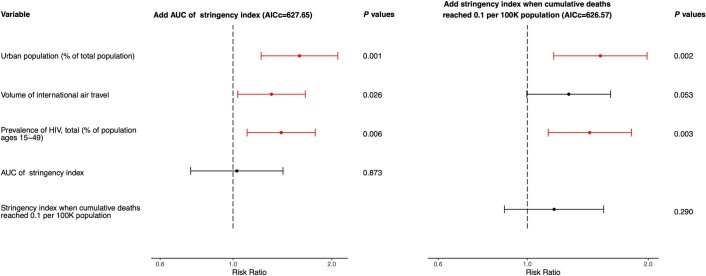
Risk ratios and 95% confidence intervals of two stringency indices for per capita mortality in the first wave in multivariable Poisson generalized linear mixed model. *n* = 42 countries. Error bars are shown. Statistically significant risk factors are in red. Exact two-sided *P* values for the Wald test are shown for each predictor, and two-sided *P* values < 0.05 were considered statistically significant.

None of the predictors in the best multivariable model was correlated with the two stringency scores (correlation coefficients < 0.6) (Extended Data Fig. 4). Again, we then re-ran the best multivariable GLMMs, once with each stringency score (Supplementary Fig. 1y,z). No stringency score was associated with the per capita mortality rate, and none reduced the AICc (Extended Data Fig. 5 and Supplementary Table 3). We explored other thresholds of cumulative per capita mortality, and all produced consistent results.

**Fig. 5:**
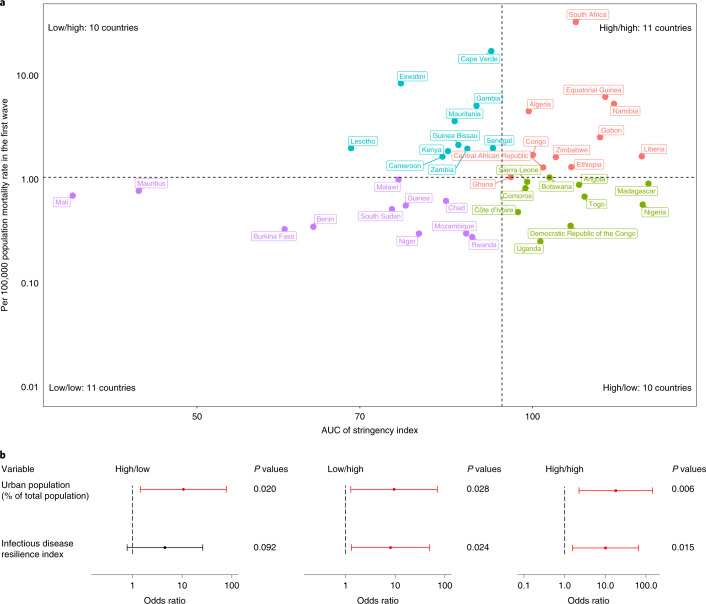
Associations with stringency index. **a**, Scatter plot for AUC of stringency index and per capita mortality rate in the first wave. Vertical axis has log_10_ scale. Dashed lines indicate median values, separating countries into four categories: high/high, high/low, low/high and low/low. **b**, Odds ratios (ORs) and 95% CIs in multivariable multinomial logistic regression model. *n* = 42 countries. Error bars are shown. Statistically significant risk factors are in red. Exact two-sided *P* values for the Wald test are shown for each predictor, and two-sided *P* values < 0.05 were considered statistically significant.

**Extended Data Fig. 6:**
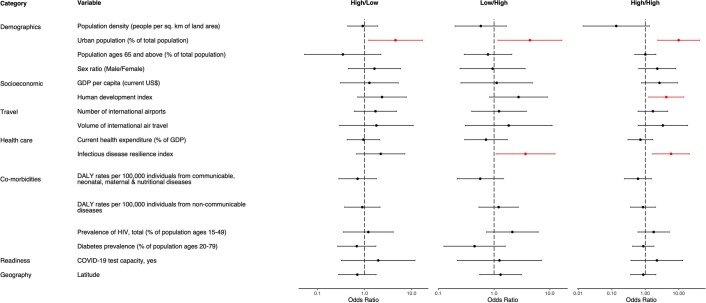
Odds ratios and 95% confidence intervals for outcome with respect to AUC of stringency index and mortality rate in the first wave in univariable multinomial logistic regression model. *n* = 42 countries. COVID-19 readiness status and number of borders were excluded from the model because there is no country with adequate COVID-19 readiness status in the reference low/low level and no country with no border in the high/high level, and putting them in the model will generate super wide 95% CIs. Wald test was used. Relative to low/low, *P* values for Wald test for percentage of urban population are 0.025 (high/low), 0.028 (low/high), and 0.002 (high/high), human development index is 0.020 (high/high), infectious disease resilience index are 0.037 (low/high) and 0.006 (high/high). Error bars are shown. Statistically significant risk factors are in red.

There were 11, 10, 10 and 11 countries in the categories of high (area under the curve (AUC) of stringency index)/high (per capita mortality), high/low, low/high and low/low, respectively (Fig. 5a). In the univariable multinomial logistic model, the percentage of urban population, infectious disease resilience index and human development index were risk factors for one or more categories relative to low/low (Extended Data Fig. 6 and Supplementary Table 4). In the multivariable multinomial logistic model, the percentage of urban population and infectious disease resilience index were risk factors for high/high, low/high and/or high/low relative to low/low (Fig. 5b). As above, we also added the three COVID-19 testing predictors into the best multivariable multinomial logistic model, and the results remained consistent (Supplementary Table 4).

### Predictors of per capita mortality during the second wave

**Extended Data Fig. 7:**
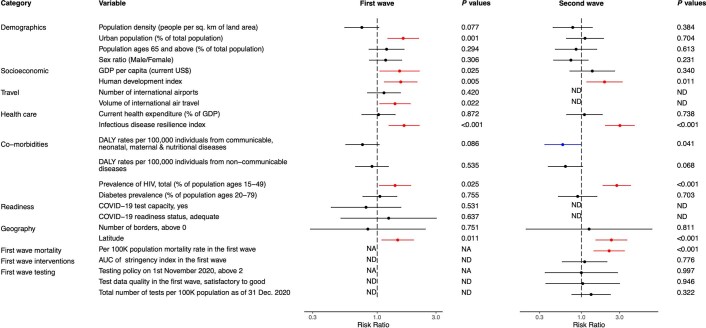
Risk ratios and 95% confidence intervals of predictors of per capita mortality in the first wave and second wave in univariable Poisson generalized linear mixed model. *n* = 42 countries. Error bars are shown. Statistically significant risk factors are in red; protective factors are in blue. Exact two-sided *P* values for the Wald test are shown for each predictor, and two-sided *P* values < 0.05 were considered statistically significant. NA, not applicable. ND, not done.

We included 42 countries and 19 predictors (Supplementary Fig. 1b–g,j–o,r,s,v–y and Fig. 1c) in the univariable GLMM for per capita mortality in the second wave. Consistent with the results for the univariable analysis of the first wave, human development index, infectious disease resilience index, prevalence of HIV and latitude were risk factors for per capita mortality in the second wave (Extended Data Fig. 7 and Supplementary Table 5). Per capita mortality rate in the first wave was also a risk factor. Disability-adjusted life years (DALYs) per 100,000 individuals from communicable, neonatal, maternal and nutritional diseases was identified as a protective factor.

## Discussion

In this study, we identified statistical predictors of the timing of the first case and the per capita mortality rates during the first and second COVID-19 pandemic waves for countries in the WHO African Region. The percentage of urban population, number of international airports, volume of pre-pandemic international air travel, COVID-19 test capacity and number of borders were predictors of the earlier detection of the first case. The percentage of urban population, volume of pre-pandemic international air travel and prevalence of HIV were risk factors for per capita mortality rate in the first pandemic wave. Stringency and timing of government restrictions were not associated with the mortality rate, but countries with higher proportions of urban population and higher infectious disease resilience scores were at increased risk of an adverse outcome, defined as either high AUC of stringency index and/or high per capita mortality. Predictors of per capita mortality rates in the two waves were broadly consistent, and per capita mortality rate in the first wave was predictive of per capita mortality rate in the second wave.

The association between laboratory capacity to test for COVID-19 cases (evaluated before the detection of COVID-19 in the WHO African Region) and earlier detection of first COVID-19 cases was expected. This result highlights the importance and urgency of ensuring adequate preparedness, especially in the earliest stages of a pandemic, noting that COVID-19 was first detected in Africa over 7 weeks after it was first detected in China^10^.

We found that countries with more international airports and a greater volume of pre-pandemic international air travel detected their first COVID-19 cases earlier, and island nations detected their first COVID-19 cases later. Flight connectivity to China was found to be a risk factor for earlier detection of COVID-19, irrespective of their preparedness status as measured by Global Health Security and Joint External Evaluation scores^11^, but genome sequencing data suggest that early cases in Africa were mainly imported from Europe and not China^12,13^.

Pre-pandemic volume of international air travel also predicts per capita mortality during the first wave. We interpret this as indicating that wider seeding of an epidemic before travel restrictions were imposed (as they were in all countries in our study) resulted in a larger epidemic.

A more urban population predicts both earlier detection of COVID-19 and a higher first wave mortality rate. Urban environments are recognized as risk factors for the transmission of respiratory pathogens in general^14^. Other studies found an association between a more urban population and the number of COVID-19 cases^15^, and that countries with higher socio-economic development, such as Belgium, United Kingdom and Italy, have higher COVID-19 mortality rates^16,17^. Countries with a more urban population and greater socio-economic development might have lower COVID-19 case fatality rates (CFRs)^15,18^. However, our study focused on per capita mortality, as CFR is heavily influenced by COVID-19 testing capability, which is highly heterogeneous across countries^3,9,19^.

We also found that a higher prevalence of HIV was associated with a higher mortality rate in the first pandemic wave. HIV has been associated with severe COVID-19 during the pandemic; a large population-based study in South Africa found that HIV doubled (HR = 2.14) the risk of COVID-19 mortality^20^. A meta-analysis of 22 studies worldwide also found that HIV-positive status was associated with an increased risk of COVID-19 mortality^21^. The underlying reasons might include a high prevalence of comorbidities in patients with HIV and severe COVID-19 and persistent immune suppression in severe COVID-19 (ref. ^20^). In our study, statistical models replacing HIV with other common comorbidities—tuberculosis (which is strongly correlated with HIV), chronic obstructive pulmonary disease, hypertensive heart disease and obesity—fitted the data less well, although it is possible that HIV status acts as a marker for a basket of these and other comorbidities. Alternatively, any link could be wholly or partially indirect if HIV prevalence is correlated with behavioral, lifestyle or socioeconomic variables not included in our analysis.

We found that stringency and timing of government restrictions were not associated with the mortality rate in the first pandemic wave. Some studies found that measures including internal ‘lockdown’ and rapid border closures were not associated with COVID-19 mortality^17,22^, whereas others found that rapid implementation of restrictions reduced COVID-19 mortality^23^. There is a complex cause-and-effect relationship between restrictions and mortality rate, and our results should not be interpreted as demonstrating that restrictions are ineffective, only that any effect is difficult to detect by a retrospective statistical analysis^3,24^. This is expected if countries that imposed more stringent restrictions more quickly did so in response to the observed or anticipated severity of their epidemic, and if differences in stringency, at best, only partially mitigated the outcome.

As the response to the pandemic is likely to be damaging in its own right (for example, through negative effects on human well-being, the economy, education and work), an alternative approach is to consider stringency as an outcome variable. The preferred outcome is a low per capita mortality rate and fewer restrictions as measured by the stringency index. Taking this approach, we found that countries were more likely to achieve a good outcome if they had a less urban population and low infectious disease resilience. Infectious disease resilience is a composite index that considers multiple factors ranging across multiple domains, including political, economic, public health, medical, demographic and disease dynamics (Supplementary Table 1). It is positively correlated with GDP per capita, the human development index, volume of international travel and prevalence of HIV, and negatively correlated with DALY rates from both communicable diseases and non-communicable diseases (Extended Data Fig. 1). This result contradicts speculation that poor countries with a low resilience would be most affected by COVID-19 (see also ref. ^11^). In Africa, more urbanized countries and those considered more resilient to infectious diseases suffered more from both the direct and indirect effects of the pandemic.

Similar results for the first and second waves suggest that there were no major shifts in the epidemiology of COVID-19 over the study period, implying no systematic differences in vulnerabilities to the two waves. There was no relationship between stringency of measures taken during the first wave and the severity of the second wave. This indicates that, regardless of the stringency and effectiveness of the government response, intrinsic differences among countries have a substantial effect on the course of national epidemics.

This study has some limitations. It is an observational study of country-level data and cannot demonstrate a direct, causal link between predictors and outcome. Effects due to unmeasured confounders might influence the results and interpretation. Statistical power is limited by sample size, so the final multivariable models include only those predictors with the strongest effects; others might have effect sizes too small to be retained in the models. Given the enormous number of combinations of predictors that could be considered, it is possible that the best fitting models were not identified. Data quality has also been raised as an issue^9^. Some, possibly substantial, under-ascertainment of COVID-19 deaths is likely in Africa, as elsewhere^25^, and could affect our findings if the degree of under-ascertainment was correlated with predictors included in our analysis. We directly addressed this issue by including in our analyses independent estimates of under-reporting of COVID-19 deaths generated by the Institute for Health Metrics and Evaluation^25^. These estimates range up to approximately 75% of COVID-19 deaths unreported (in Burkina Faso, Nigeria and the Democratic Republic of the Congo). The WHO definition of a COVID-19 death does not require a positive test result, but it is possible that ascertainment is influenced by testing capacity. However, our main results are robust to inclusion of indicators of testing effort in our statistical models, although we note that test volume data were not collected over exactly the same time period.

The stringency variable is a composite index of government policies, reflecting that many countries implemented measures as a package. Not all policies are expected to have equal effect, and a wide range of combinations of measures was implemented across the region. We validated the stringency index by comparison with Google mobility data. We found a strong association, indicating that the index is related to real-world behavior by at least a subset of the population. However, the association weakened over time, as has been reported elsewhere^26^.

Our study had several strengths. We considered countries from a single WHO region; these should be more comparable in terms both of data on predictors and of COVID-19 epidemiology. We restricted our analysis to outcome variables judged to be most reliably estimated—date of first case and mortality—while correcting for under-reporting/under-ascertainment. The evident plausibility of the results of our date of first case analysis improves confidence that the predictor and outcome data are fitted for purpose.

In conclusion, we identified risk factors associated with poor direct and indirect outcomes of the first two waves of the COVID-19 pandemic in the WHO African Region countries. Our key finding is that countries that were assumed to be better prepared and better equipped to respond to the pandemic were also the most vulnerable to it. These data should be taken into account in future pandemic preparedness planning for WHO African Region countries.

## Methods

### Ethics statement

Ethics approval was not required for this study as the data used in the study were at the country level, and the study is observational.

### Study design and study area

**Extended Data Fig. 8:**
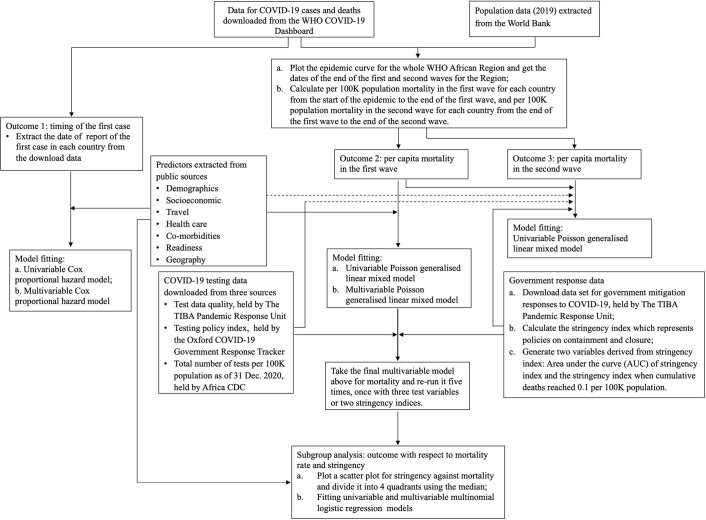
Flow diagram.

We performed a region-wide, country-based observational study (Extended Data Fig. 8) that included all 47 Member States of the WHO African Region. The WHO African Region has a total population of 1,019,922,000, with the median age varying from 15.0 years in Niger to 34.6 years in Mauritius^27^. About 50% of the population in the WHO African Region lack access to essential medicines^28^. Globally, 22 of the 25 countries regarded as most vulnerable to infectious diseases are in sub-Saharan Africa^29^.

We extracted data for daily cases and deaths for each country in the region and calculated the following three outcomes: timing of the first case and per capita mortality rates in the first and second waves. Predictors relating to demographics, socioeconomics, travel, healthcare, comorbidities, readiness and geography were extracted from public data sources. The ratio of total COVID-19 mortality to reported COVID-19 mortality was obtained from the Institute for Health Metrics and Evaluation^25^. The COVID-19 test data quality and the government response data were collected by the Tackling Infections to Benefit Africa (TIBA) Pandemic Response Unit. COVID-19 testing policy data were taken from the Oxford COVID-19 Government Response Tracker (OxCGRT). Total numbers of tests per capita were collected by the Africa Centres for Disease Control and Prevention (CDC)^3^. Statistical models were fitted to evaluate the relationships among the three outcomes and predictors. We also ran a secondary analysis for the outcomes per capita mortality in the first wave and stringency index.

The start date of the analysis was set as 25 February 2020 when the first case was reported from the WHO African Region (in Algeria). We collated values of predictor variables as close to this date as possible.

### Outcomes

Our first outcome—the timing of the first case—refers to the day on which the first official laboratory-confirmed COVID-19 case/cases was/were reported to the WHO (Fig. 1a), largely based on case definitions defined by the WHO^30^.

Our other outcomes are the total deaths per 100,000 population (per capita mortality rate) during the first and second waves, adjusted for under-reporting where appropriate (see below). According to international guidelines for certificate and coding of COVID-19 as cause of death^31^, a death due to COVID-19 is defined for surveillance purposes as a death resulting from a clinically compatible illness, in a probable or confirmed COVID-19 case, unless there is a clear alternative cause of death that cannot be related to COVID disease (for example, trauma). There should be no period of complete recovery from COVID-19 between illness and death. A death due to COVID-19 might not be attributed to another disease (for example, cancer) and should be counted independently of pre-existing conditions that are suspected of triggering a severe course of COVID-19.

The pandemic curve for daily new deaths for the whole WHO African Region was plotted by using 21-d kernel smoothing using the Nadaraya–Watson estimator (Fig. 1b). Kernel smoothing is a common non-parametric method for revealing trends in curves. The Nadaraya–Watson estimator can be seen as a weighted average using kernel as weighting functions, and a higher weight was assigned to daily new deaths closer to the target date^32^. We chose the date with the first minimum daily new deaths (31 October 2020) as the end of the first wave and the date of the second minimum daily new deaths (14 March 2021) as the end of the second wave, and we calculated per capita mortality rate in each wave for each country.

Data on COVID-19 cases and deaths for all 47 Member States in the WHO African Region were taken from the WHO COVID-19 Dashboard^33^. The data include daily new cases, cumulative cases, daily new deaths and cumulative deaths.

### Predictors

A set of predictors considered likely to affect the timing of the first case and the per capita mortality rate were collected and included as explanatory variables. The definition, reasons for including the predictor, time range, details of missing data and data sources are reported in Supplementary Table 1. Predictors were classified in nine categories: demographics, socioeconomics, travel, healthcare, comorbidities, readiness, geography, COVID-19 testing and interventions. Demographic and socioeconomic variables might predict both vulnerability to severe disease (for example, by age) and transmission potential (for example, urban versus rural populations)^19,34^. Healthcare, readiness and COVID-19 testing variables might predict the capability to detect and/or treat cases^17,35^. Travel and the number of shared borders are likely to affect the imported cases from neighboring countries^36^. Comorbidities are related to vulnerability to dying from infection^16^. Latitude is related to climate, which might affect transmission rates^37^.

Data on COVID-19 testing were obtained from four sources. Testing effort was extracted from a recent report of the COVID-19 pandemic in Africa up to the end of December 2020 (ref. ^3^). The predictor variable was total number of tests divided by per 100,000 population. Testing policy index data were collected by OxCGRT, which records government policy on access to testing. The ordinal scores are shown in Supplementary Table 1, and we calculated days with testing policy index ≥2 during the first wave (25 February to 31 October 2020). Testing policy index at the start of the second wave on 1 November 2020 was used as a baseline predictor for per capita mortality in the second wave. A test data quality index up to 31 October was generated by the TIBA Pandemic Response Unit and was placed into four categories (no data, basic, satisfactory and good; Supplementary Tables 1 and 6). Details of data collection are given in the TIBA COVID-19 testing report^38^. Estimated ratios of total COVID-19 mortality to reported COVID-19 mortality were obtained from the Institute for Health Metrics and Evaluation (Supplementary Table 1)^25^.

Government response data were collected by the TIBA Pandemic Response Unit. Details of data collection are given in the TIBA COVID-19 mitigation policies report^39^. All mitigation responses fall into five categories and 14 subcategories (Supplementary Table 7). Normalized strictness scores were devised for each of the 14 subcategories. Based on these normalized strictness scores, the stringency index representing policies on containment and closure were calculated using a method developed by OxCGRT^40^, which is by averaging the normalized strictness values of 12 subcategories of measures, excluding all governance and socio-economic measures and surveillance and testing from public health measures.

Two variables related to the stringency index were generated: AUC of stringency index scores from 25 February to 31 October 2020 and stringency index score when cumulative mortality reached 0.1 per 100,000 population during the first wave. Alternative thresholds ranging from 0.001 to 0.2 were also explored for validation.

**Extended Data Fig. 9:**
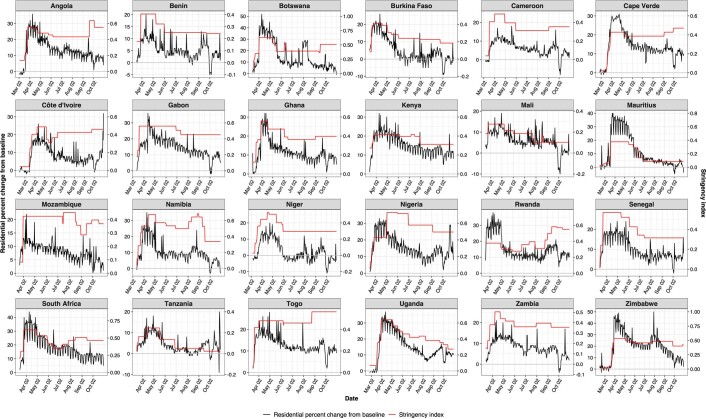
Residential percent change from baseline and stringency index over time in 24 countries of the WHO African Region. Time range is from first countermeasure implemented in each country in response to COVID-19 up to 31 October 2020. Y1 axis represents residential percent change from baseline (Jan 3 – Feb 6, 2020) in Google mobility data (in black). Y2 axis represents stringency index calculated from government response data collected by the TIBA Pandemic Response Unit (in red, see Methods).

Google mobility data (https://www.google.com/covid19/mobility/) available for 25 WHO African Region Member States were used to validate the data for the stringency. Details of Google mobility data are included in the TIBA COVID-19 mitigation policies report^39^. The residential percent change of mobility was used to validate stringency index for the following reasons: (1) the residential category has a high correlation coefficient with the other five categories of mobility; (2) the location accuracy and the understanding vary less across regions than other categories, so the comparison among countries will cause less bias; and (3) the intention of many mitigation response measures is to encourage people to stay in their residence. As of 15 November 2020, 24 of 47 WHO African countries had mobility data for the residential category. Time series plots of stringency index against residential mobility are shown in Extended Data Fig. 9. We used a generalized additive mixed model to estimate the relationships between the stringency index and residential mobility over time. We fitted the residential mobility as a spline function of stringency index *s*(*Stringency index*) and a spline function of day of the year *s*(*doy*), which was used to control for the temporal trend. The temporal relationship between residential mobility and stringency index can be different among countries, so we also introduced a spline function of country *s*(*country, bs* = ‘*re*’) as random intercepts and country and day of the year (*country*, *doy*, *bs* = ‘*re*’) as random slopes. The model was expressed as follows.

$$\begin{array}{rcl}g(Y_{ij}) & =& s(Stringency\,index) + s(doy) + s(country,bs = {\,}^{\prime} re^{\prime} )\\ && + s(country,doy,bs = {\,}^{\prime}re^{\prime} ) + \varepsilon _{ij}\end{array}$$

where *Y*_*ij*_ denoted the residential mobility for the *i*th day in the *j*th country, and *ε*_*ij*_ is the random noise. *s*() indicated penalized spline function. *bs* = ‘*re*’ indicated that the basis function is a random effect structure (basis coefficients are penalized by a ridge penalty to control the degree of smoothness). We used the default parameter settings from the R package mgcv for penalized spline function.

### Statistical methods

All 47 Member States were included in the model for the timing of the first case, but the number of Member States included in the model for per capita mortality in two waves depended on the completeness of the data. The epidemic curves for both daily cases and deaths in each country within the WHO African Region were plotted to evaluate the completeness of the data. The government of the United Republic of Tanzania stopped reporting COVID-19 cases/deaths from 8 May and, therefore, was excluded.

For predictors, the most recent available data were used—and no earlier than 2010. If one predictor had missing values, one column of binary indicator was added showing which country has missing data and which has not, and both the raw data and the indicator were included in the model. All predictors used had data available for at least 90% of countries.

Spearman’s rank correlation was used to test for a correlation among predictors. Predictors with a correlation coefficient greater than 0.6 were not included in the same multivariable model.

Cox proportional hazards regression models were used to determine HRs and 95% CIs for individual predictors of timing of the first case. A univariable model was fitted first. Only predictors quantified on or before the start date were included in this analysis. Comorbidity data were excluded, as there is no a priori expectation that these would be predictors. COVID-19 test capacity, COVID-19 readiness status and the number of borders entered the model as binary variables where ‘no’, ‘limited and moderate’ and ‘no border’ were set as the reference levels, respectively. For COVID-19 readiness status, we combined ‘limited’ and ‘moderate’ into one single level—‘limited and moderate’, because only two countries were at the ‘limited’ level (Supplementary Fig. 1q). Three countries (Cape Verde, Mauritius and Seychelles) with unknown COVID-19 readiness status were also included in the ‘limited and moderate’ level. Other variables entered the model as continuous variables, and all continuous variables were standardized before entering the model by subtracting the mean and dividing by the standard deviation. Variables with *P* values less than 0.2 were considered for inclusion in a multivariable model. If multiple variables with *P* values less than 0.2 were highly correlated (correlation coefficient greater than 0.6), only one variable was selected each time to enter the multivariable model. The multivariable model with the lowest AICc was taken as the best model^41^, but models with +2 AICc scores were also retained.

We used a GLMM with a Poisson error distribution to identify predictors of per capita mortality rate in the first wave. We used the reported deaths times the ratio of total COVID-19 mortality to reported COVID-19 mortality (Supplementary Fig. 1t) as the outcome, population size as an offset and country as a random effect. The RRs and 95% CIs were calculated. Five countries (the United Republic of Tanzania having incomplete data, Burundi, Eritrea and Seychelles being clear outliers and Seychelles and São Tomé and Príncipe having missing data for HIV prevalence) were excluded (also for the multinomial logistic model below for outcome with respect to per capita mortality in the first wave and stringency). Days with testing policy index ≥2 entered the model as a binary variable (using median as the cutoff) where ‘below median’ was set as the reference level. Three countries (Guinea Bissau, Equatorial Guinea and Comoros) with missing days with testing policy index were included in the ‘below median’ level. We treated test data quality as binary, combining no data and basic data to the lower level (reference level), and satisfactory data and good data to the higher level. Univariable models and the best multivariable model were fitted using the same approach as for the timing of the first case. We then added the two stringency scores (AUC of stringency index in Supplementary Fig. 1y and stringency index when cumulative deaths reached 0.1 per 100,000 population in Supplementary Fig. 1z) to the best multivariable model and checked for significantly improved model fit (lower AICc). We first estimated the correlations between the two stringency scores and the set of selected predictors in the best multivariable model, using the Spearman rank correlation test. Then, we took the best multivariable model and re-ran it by adding each stringency score. Again, only stringency scores with correlation coefficients less than 0.6 with the set of selected predictors were included in the multivariable model. We repeated this exercise for the three testing variables—that is, adding days with testing policy index ≥2 (Supplementary Fig. 1u), test data quality (Supplementary Fig. 1w) and tests per capita (Supplementary Fig. 1x) to the best multivariable model for per capita mortality in the first wave and asking whether the result was consistent after adjusting for COVID-19 testing.

We carried out a secondary analysis using the original set of predictors of COVID-19 mortality in the first wave to predict an outcome combining per capita mortality in the first wave and stringency index. In this analysis, countries were placed into four groups based on the medians of total per capita mortality in the first wave and of the AUC of stringency index (high stringency/high mortality, high stringency/low mortality, low stringency/high mortality and low stringency/low mortality). Multinomial logistic regression was used to estimate the relationship between these outcomes and the set of predictors, and the ORs and 95% CIs were calculated. Univariable models and the best multivariable model were fitted using the same approach as for the first wave mortality rate. Low stringency/low mortality was set as the reference level. COVID-19 readiness status and number of borders were excluded from the model because no country in the low/low level had adequate COVID-19 readiness status, and there was no island nation in the high/high level.

For the second wave mortality rate analysis, we fitted only the univariable model using the same approach as for first wave mortality rate. We dropped predictors related to travel and readiness, given that these pre-pandemic predictors cannot represent the baseline level at the start of the second wave. We added per capita mortality in the first wave (Fig. 1c) and testing policy index on 1 November 2020 (Supplementary Fig. 1v) as two new predictors. Testing policy index on 1 November 2020 entered the model as a binary predictor where ‘below 2’ was set as the reference level. AUC of stringency in the first wave (Supplementary Fig. 1y), test data quality in the first wave (Supplementary Fig. 1w) and tests per capita as of 31 December 2020 (Supplementary Fig. 1x) were considered as predictors of second wave mortality rate, respectively.

R version 3.6.3 (R Foundation for Statistical Computing) was used in all statistical analyses. R packages used for model fitting included survival, lme4, nnet and mgcv. A two-sided *P* value < 0.05 was regarded as statistically significant. The raw African shapefile used in the study was obtained from Data and Maps for ArcGIS (formerly Esri Data & Maps, https://www.arcgis.com/home/group.html?id=24838c2d95e14dd18c25e9bad55a7f82#overview) (see the permission for use in Supplementary Table 8). Further information on predictors^42–53^ is given as Supplementary Information.

### Reporting Summary

Further information on research design is available in the Nature Research Reporting Summary linked to this article.

## Online content

Any methods, additional references, Nature Research reporting summaries, source data, extended data, supplementary information, acknowledgements, peer review information; details of author contributions and competing interests; and statements of data and code availability are available at 10.1038/s41591-021-01491-7.

## Supplementary information

Supplementary Information Supplementary Fig. 1 and Supplementary Tables 1–8

Reporting Summary

## Data Availability

Data on COVID-19 cases and deaths were from the WHO COVID-19 Dashboard (https://covid19.who.int/info/). Data sources for predictors included the World Bank, the United Nations, the Rand Corporation, Our World in Data, the WHO Region Office for Africa, Data and Maps for ArcGIS (formerly Esri Data & Maps), the Institute for Health Metrics and Evaluation, the Oxford COVID-19 Government Response Tracker and the Africa CDC (links are provided in Supplementary Table 1). All data are available via figshare at 10.6084/m9.figshare.15022503.

## Code availability

All code is available via figshare at 10.6084/m9.figshare.15022503.

## References

[1] World Health Organization. Weekly epidemiological update on COVID-19. https://www.who.int/emergencies/diseases/novel-coronavirus-2019/situation-reports (2021).

[2] Hsiang S (2020). The effect of large-scale anti-contagion policies on the COVID-19 pandemic. Nature.

[3] Salyer SJ (2021). The first and second waves of the COVID-19 pandemic in Africa: a cross-sectional study. Lancet.

[4] Zheng Z (2020). Risk factors of critical & mortal COVID-19 cases: a systematic literature review and meta-analysis. J. Infect..

[5] Ji Y, Ma Z, Peppelenbosch MP, Pan Q (2020). Potential association between COVID-19 mortality and health-care resource availability. Lancet Glob. Health.

[6] Kandel N, Chungong S, Omaar A, Xing J (2020). Health security capacities in the context of COVID-19 outbreak: an analysis of International Health Regulations annual report data from 182 countries. Lancet.

[7] Brauer M, Zhao JT, Bennitt FB, Stanaway JD (2020). Global access to handwashing: implications for COVID-19 control in low-Income countries. Environ. Health Perspect..

[8] Walker PGT (2020). The impact of COVID-19 and strategies for mitigation and suppression in low- and middle-income countries. Science.

[9] Rice BL (2021). Variation in SARS-CoV-2 outbreaks across sub-Saharan Africa. Nat. Med..

[10] Zhu N (2020). A novel coronavirus from patients with pneumonia in China, 2019. N. Engl. J. Med..

[11] Haider N (2020). The Global Health Security index and Joint External Evaluation score for health preparedness are not correlated with countries’ COVID-19 detection response time and mortality outcome. Epidemiol. Infect..

[12] Lu, L., Lycett, S., Ashworth, J., Mutapi, F. & Woolhouse, M. What are SARS-CoV-2 genomes from the WHO Africa region member states telling us? *BMJ Glob. Health* **6**, e004408 (2021). 10.1136/bmjgh-2020-004408 PMC7798429 33419930

[13] Ngoi JM (2021). Genomic analysis of SARS-CoV-2 reveals local viral evolution in Ghana. Exp. Biol. Med. (Maywood).

[14] Norwegian Institute of Public Health. Urbanization and preparedness for outbreaks with high-impact respiratory pathogens. https://apps.who.int/gpmb/assets/thematic_papers_2020/tp_2020_4.pdf (2020).

[15] Li M (2020). Identifying novel factors associated with COVID-19 transmission and fatality using the machine learning approach. Sci. Total Environ..

[16] Hashim MJ, Alsuwaidi AR, Khan G (2020). Population risk factors for COVID-19 mortality in 93 countries. J. Epidemiol. Glob. Health.

[17] Chaudhry R, Dranitsaris G, Mubashir T, Bartoszko J, Riazi S (2020). A country level analysis measuring the impact of government actions, country preparedness and socioeconomic factors on COVID-19 mortality and related health outcomes. EClinicalMedicine.

[18] Asfahan, S. et al. Early trends of socio-economic and health indicators influencing case fatality rate of COVID-19 pandemic. *Monaldi Arch. Chest Dis.* **90**, 10.4081/monaldi.2020.1388 (2020). 10.4081/monaldi.2020.1388 32696629

[19] Lawal Y (2021). Africa’s low COVID-19 mortality rate: a paradox?. Int. J. Infect. Dis..

[20] Boulle, A. et al. Risk factors for COVID-19 death in a population cohort study from the Western Cape Province, South Africa. *Clin. Infect. Dis*. 10.1093/cid/ciaa1198 (2020). 10.1093/cid/ciaa1198 PMC7499501 32860699

[21] Ssentongo P (2021). Epidemiology and outcomes of COVID-19 in HIV-infected individuals: a systematic review and meta-analysis. Sci. Rep..

[22] Leffler CT (2020). Association of country-wide coronavirus mortality with demographics, testing, lockdowns, and public wearing of masks. Am. J. Trop. Med. Hyg..

[23] Fountoulakis KN, Fountoulakis NK, Koupidis SA, Prezerakos PE (2020). Factors determining different death rates because of the COVID-19 outbreak among countries. J. Public Health (Oxf.).

[24] Woolhouse M (2011). How to make predictions about future infectious disease risks. Philos. Trans. R. Soc. Lond. B Biol. Sci..

[25] Institute for Health Metrics and Evaluation. Estimation of total mortality due to COVID-19. http://www.healthdata.org/special-analysis/estimation-excess-mortality-due-covid-19-and-scalars-reported-covid-19-deaths (2021).

[26] Nouvellet P (2021). Reduction in mobility and COVID-19 transmission. Nat. Commun..

[27] World Health Organization. Global Health Observatory data repository. https://apps.who.int/gho/data/node.home (2020).

[28] Kirigia JM, Sambo HB, Sambo LG, Barry SP (2009). Economic burden of diabetes mellitus in the WHO African region. BMC Int. Health Hum. Rights.

[29] Moore, M., Gelfeld, B. & Okunogbe, A. Identifying future disease hot spots: Infectious Disease Vulnerability Index. https://www.rand.org/pubs/research_reports/RR1605.html (2016). 10.7249/rhq-06-03-05 PMC5568150 28845357

[30] World Health Organization. WHO COVID-19: Case Definitions. https://apps.who.int/iris/handle/10665/337834 (2020).

[31] World Health Organization. International guidelines for certificate and classification (coding) of COVID-19 as cause of death. https://www.who.int/classifications/icd/Guidelines_Cause_of_Death_COVID-19.pdf (2020).

[32] Hazelton, M. L. Kernel Smoothing. In *Wiley StatsRef: Statistics Reference Online* (eds Balakrishnan, N. et al.) 10.1002/9781118445112.stat06538 (2014).

[33] World Health Organization. WHO Coronavirus Disease (COVID-19) Dashboard. https://covid19.who.int/info/ (2021). 37184163

[34] Hradsky O, Komarek A (2021). Demographic and public health characteristics explain large part of variability in COVID-19 mortality across countries. Eur. J. Public Health.

[35] Okeahalam C, Williams V, Otwombe K (2020). Factors associated with COVID-19 infections and mortality in Africa: a cross-sectional study using publicly available data. BMJ Open.

[36] Chinazzi M (2020). The effect of travel restrictions on the spread of the 2019 novel coronavirus (COVID-19) outbreak. Science.

[37] Tzampoglou, P. & Loukidis, D. Investigation of the importance of climatic factors in COVID-19 worldwide intensity. *Int. J. Environ. Res. Public Health* **17**, 7730 (2020). 10.3390/ijerph17217730 PMC7660112 33105818

[38] TIBA Pandemic Response Unit. COVID-19 testing report for WHO Africa Region. https://tiba-partnership.org/tiba/sites/sbsweb2.bio.ed.ac.uk.tiba/files/pdf/TIBA-PRU%20Testing%20Data%2022.10.2020.pdf (2020).

[39] TIBA Pandemic Response Unit. COVID-19 mitigation policies by governments and changes in behaviour across WHO Africa Region. https://tiba-partnership.org/tiba/sites/sbsweb2.bio.ed.ac.uk.tiba/files/pdf/COVID-19%20mitigation%20policies%20by%20governments%20and%20changes%20in%20behaviour%20across%20WHO%20Africa%20Region.pdf (2020).

[40] Hale, T., Webster, S., Petherick, A., Phillips, T. & Kira, B. Oxford COVID-19 Government Response Tracker. https://www.bsg.ox.ac.uk/research/research-projects/coronavirus-government-response-tracker (2020). 10.1038/s41562-021-01079-8 33686204

[41] Emiliano PC, Vivanco MJF, de Menezes FS (2014). Information criteria: how do they behave in different models?. Comput. Stat. Data Anal..

[42] Arsalan, M., Mubin, O., Alnajjar, F. & Alsinglawi, B. COVID-19 global risk: expectation vs. reality. *Int. J. Environ. Res. Public Health* **17**, 5592 (2020). 10.3390/ijerph17155592 PMC7432363 32756513

[43] Takahashi T (2020). Sex differences in immune responses that underlie COVID-19 disease outcomes. Nature.

[44] Peckham H (2020). Male sex identified by global COVID-19 meta-analysis as a risk factor for death and ITU admission. Nat. Commun..

[45] Pana TA (2021). Country-level determinants of the severity of the first global wave of the COVID-19 pandemic: an ecological study. BMJ Open.

[46] Mena, G. E. et al. Socioeconomic status determines COVID-19 incidence and related mortality in Santiago, Chile. *Science* **372**, eabg5298 (2021). 10.1126/science.abg5298 PMC8158961 33906968

[47] Azarpazhooh MR (2020). COVID-19 pandemic and burden of non-communicable diseases: an ecological study on data of 185 countries. J. Stroke Cerebrovasc. Dis..

[48] Khan JR, Awan N, Islam MM, Muurlink O (2020). Healthcare capacity, health expenditure, and civil society as predictors of COVID-19 case fatalities: a global analysis. Front. Public Health.

[49] Vadlamannati KC, Cooray A, de Soysa I (2021). Health-system equity, egalitarian democracy and COVID-19 outcomes: an empirical analysis. Scand. J. Public Health.

[50] Tian W (2020). Predictors of mortality in hospitalized COVID-19 patients: a systematic review and meta-analysis. J. Med. Virol..

[51] Tamuzi JL (2020). Implications of COVID-19 in high burden countries for HIV/TB: a systematic review of evidence. BMC Infect. Dis..

[52] Sanyaolu, A. et al. Comorbidity and its impact on patients with COVID-19. *SN Compr. Clin. Med.*10.1007/s42399-020-00363-4 (2020). 10.1007/s42399-020-00363-4 PMC7314621 32838147

[53] Rahman, M. et al. A global analysis on the effect of temperature, socio-economic and environmental factors on the spread and mortality rate of the COVID-19 pandemic. *Environ. Dev. Sustain.*10.1007/s10668-020-01028-x (2020). 10.1007/s10668-020-01028-x PMC7538192 33041644

