# Supplementary material for: Predictors of COVID-19 epidemics in countries of the World Health Organization African Region
Source: Nat Med. 2021 Sep 3;27(11):2041–7. doi: 10.1038/s41591-021-01491-7 (PMC8604723; doi:10.1038/s41591-021-01491-7)
Supplement: Supplementary file 1 — Supplementary Fig. 1 and Supplementary Tables 1–8 [file 41591_2021_1491_MOESM1_ESM.pdf]

nature portfolio

https://doi.org/10.1038/s41591-021-01491-7

Supplementary information

Predictors of COVID-19 epidemics in countries of the World Health Organization African Region

In the format provided by the
authors and unedited

Supplementary Fig. 1 Distribution of predictors

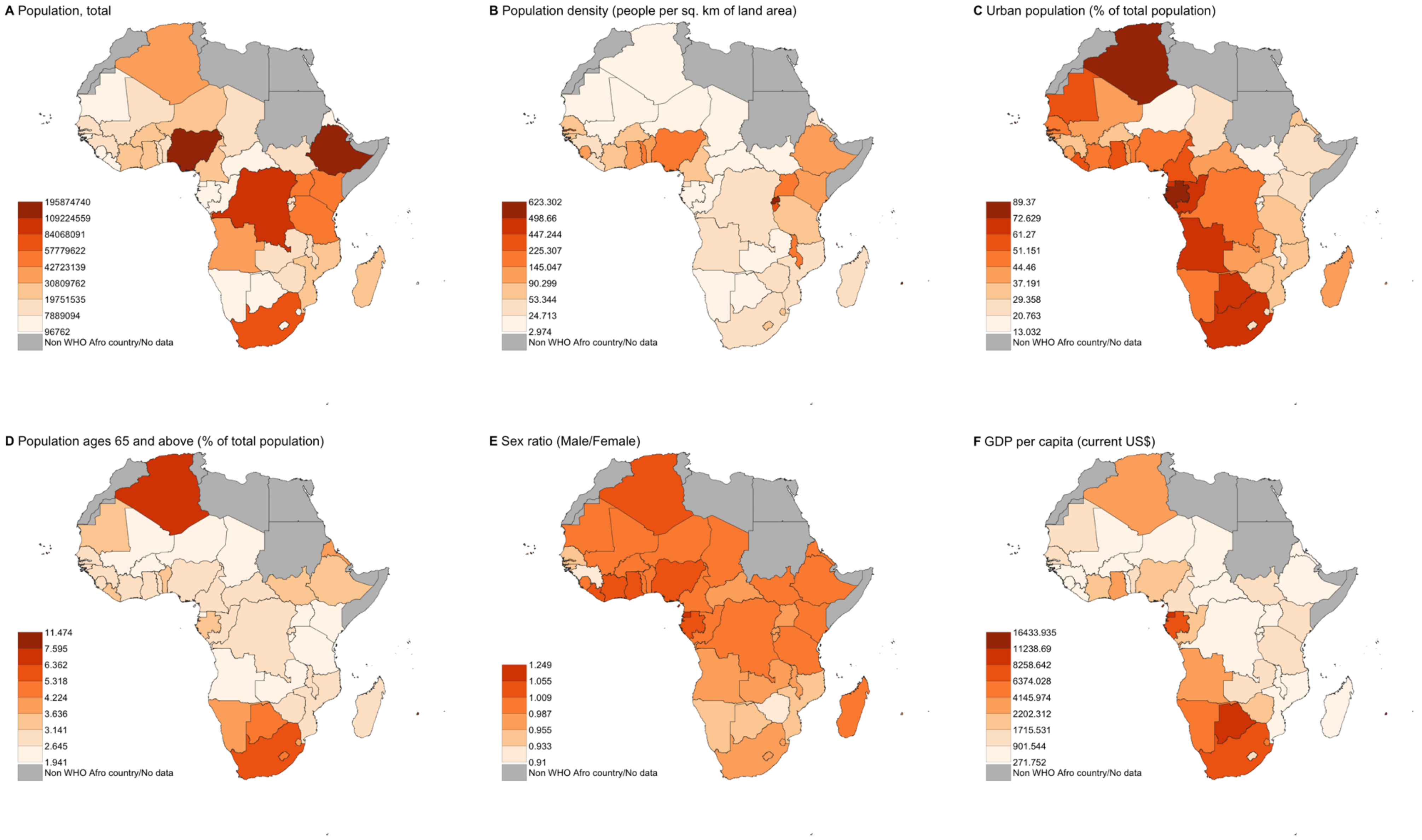

Supplementary Fig. 1 (continued 1)

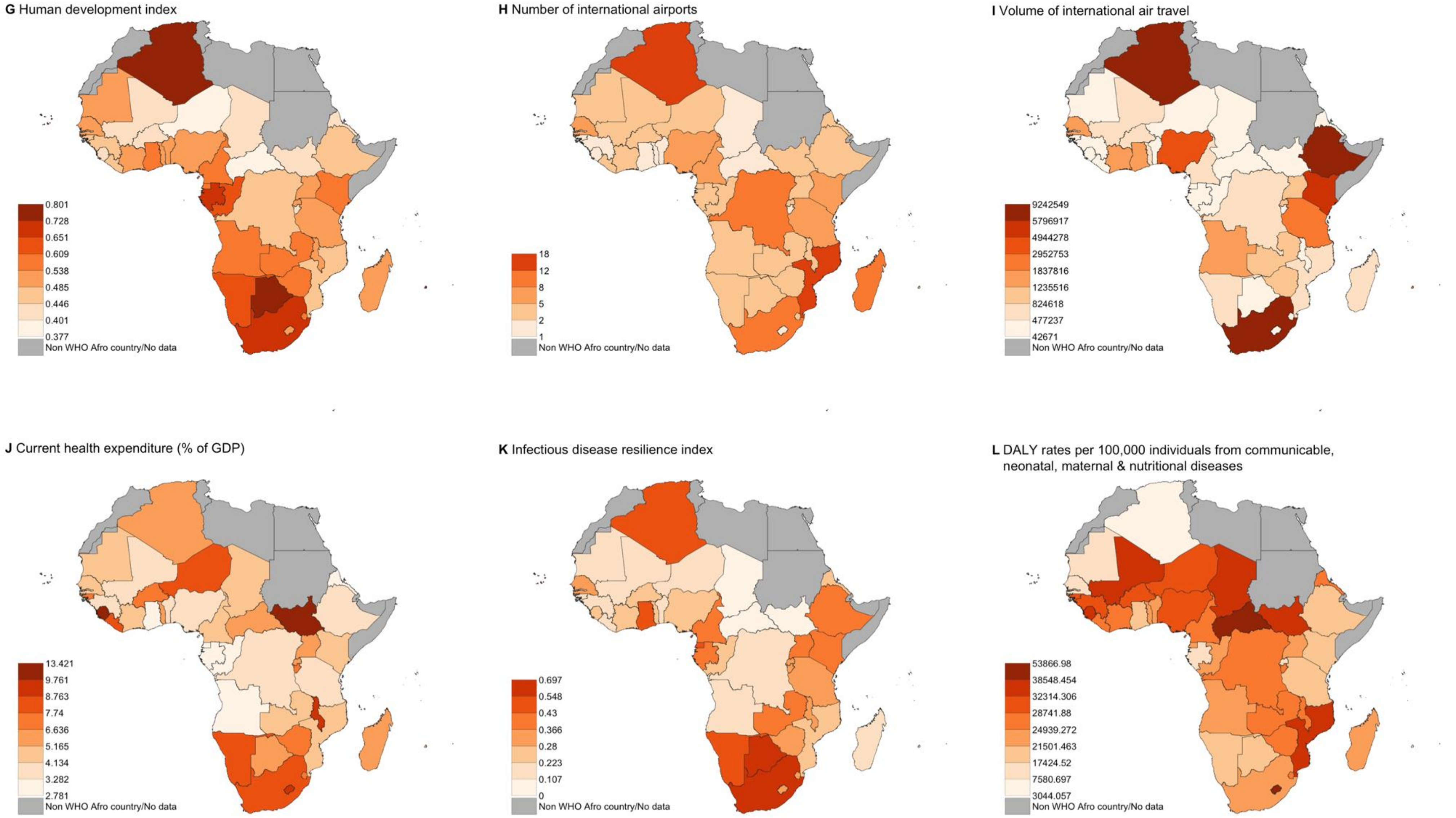

Supplementary Fig. 1 (continued 2)

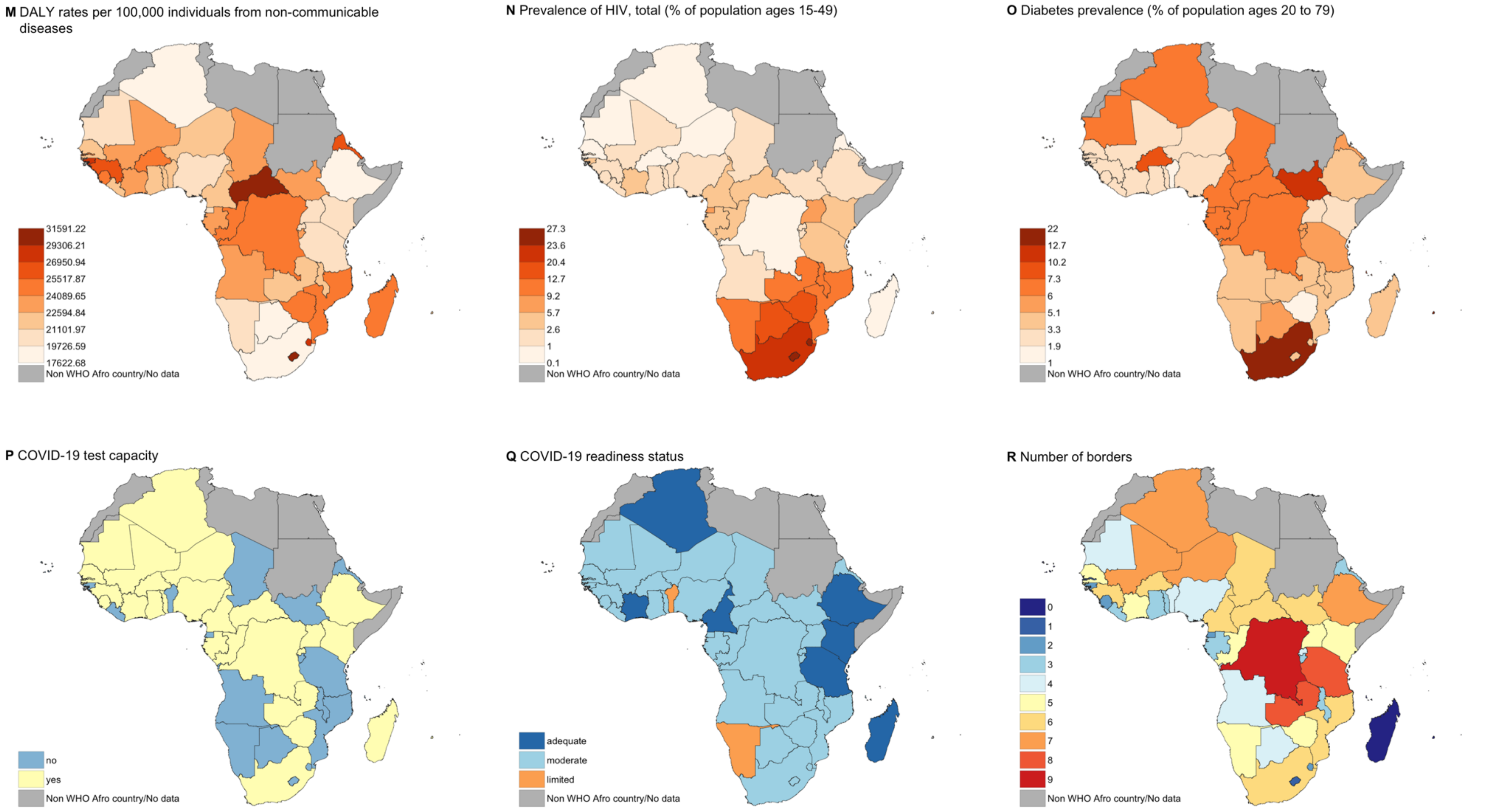

Supplementary Fig. 1 (continued 3)

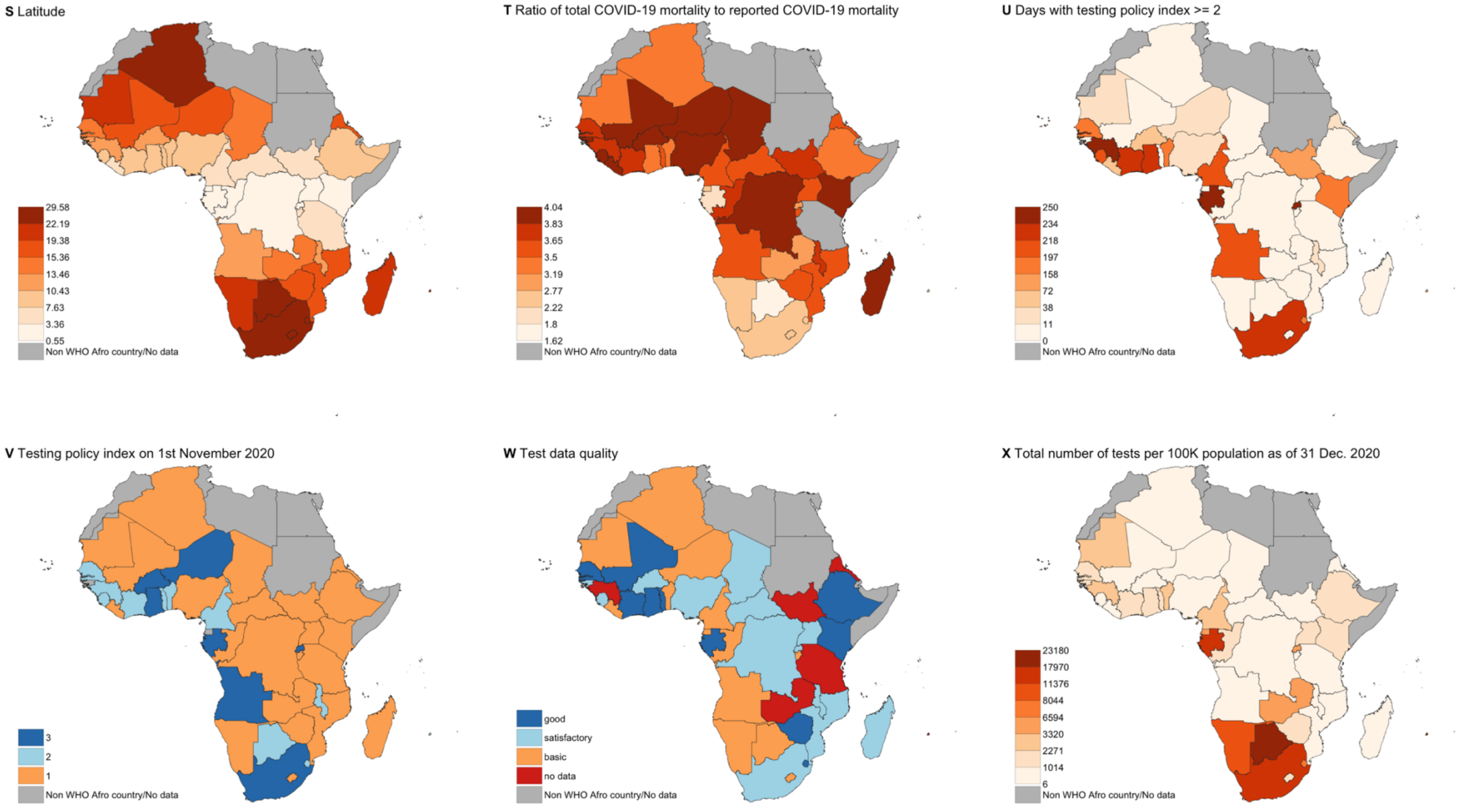

Supplementary Fig. 1 (continued 4)

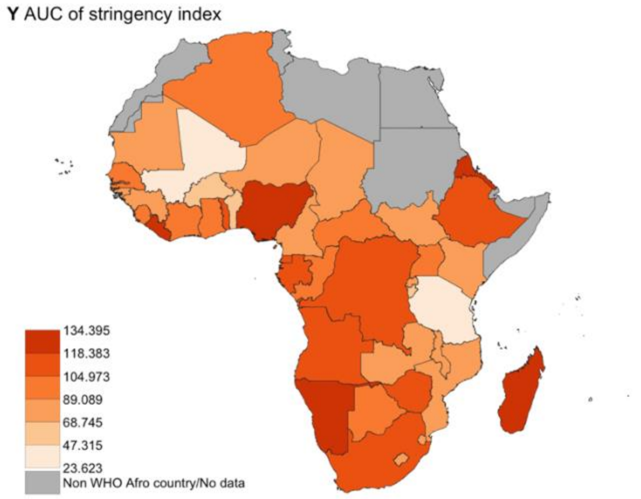

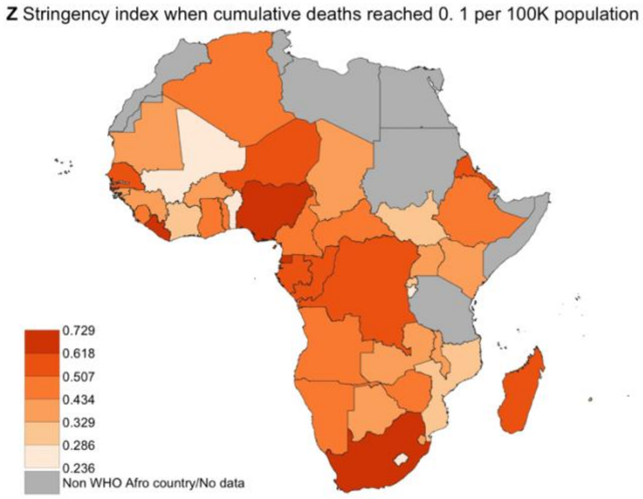

Supplementary Table 1. Predictors of interest

Category	Definition from data source	Reasons for including the predictor	Time range	Source
**Demographics**	1. Population, total<br>Population, total is based on the de facto definition of population, which counts all residents regardless of legal status or citizenship – except for refugees not permanently settled in the country of asylum, who are generally considered part of the population of their country of origin. The values shown are midyear estimates.	COVID-19 morbidities and mortalities are derived from the entire human population, so scale with population size.	2018, except that Eritrea data from 2011	The World Bank https://data.worldbank.org/indicator
	2. Population density (people per sq. km of land area)<br>Midyear population divided by land area in square kilometres. Definition of population is as shown above. Land area is a country's total area, excluding area under inland water bodies, national claims to continental shelf, and exclusive economic zones. In most cases the definition of inland water bodies includes major rivers and lakes.	Population density and the proportion of urban population may directly affect the transmission dynamics of SARS-CoV-2, causing more morbidities and mortalities in countries with denser population/more urban population[34].	2018, except that Eritrea data from 2011. Data of South Sudan was missing and was added manually, using data of total population above and land area from the government of the Republic of South Sudan (http://www.goss-online.org/about.html).	
	3. Urban population (% of total population)<br>People living in urban areas as defined by national statistical offices. The data are collected and smoothed by United Nations Population Division.		2018, except that Eritrea data from 2011	
	4. Population ages 65 and above (% of total population)<br>Population ages 65 and above as a percentage of the total population. Population is based on the de facto definition of population, which counts all residents regardless of legal status or citizenship.	The elderly has been found to be more vulnerable to severe/fatal COVID-19[19,22,34,37,42].	2018, except that Eritrea data from 2011	
	5. Sex ratio (Male/Female)<br>Ratio of male population count to female population count.	Men have been found to be affected by COVID-19 with more severe symptoms and higher mortality than women[43,44].	2018, except that Eritrea data from 2011	
**Socioeconomic**	1. GDP per capita (current US$)<br>Gross domestic product divided by midyear population. GDP is the sum of gross value added by all resident producers in the economy plus any product taxes and minus any subsidies not included in the value of the products. It is calculated without making deductions for depreciation of fabricated assets or for depletion and degradation of natural resources. Data are in current U.S. dollars.	Socioeconomic level may indirectly affect the COVID-19 pandemic in multiple ways through associations with lifestyles and behaviour, physical environment and the availability of health care[16,45,46].	2018, except that Eritrea data from 2011, and South Sudan data from 2015	The World Bank https://data.worldbank.org/indicator
	2. Human development index<br>Human development index is a summary measure of average achievement in key dimensions of human development: a long and healthy life, being knowledgeable and have a decent standard of living.		2018	United Nations http://hdr.undp.org/en/data

**Travel**	1. Number of international airports<br>Total number of international airports within each country, extracted from a repository of air traffic flow.	Travel-related factors, as a proxy of global connections, are likely to affect the volume of imported COVID-19 cases so seeding an earlier and larger epidemic[36,45,47].	2019	The World Bank<br>https://datacatalog.worldbank.org/dataset/global-airports
	2. Volume of international air travel<br>Total seats from the most recent year (2019) for all airports with international air travel within each country.			
**Health care**	1. Current health expenditure (% of GDP)<br>Level of current health expenditure expressed as a percentage of GDP. Estimates of current health expenditures include healthcare goods and services consumed during each year. This indicator does not include capital health expenditures such as buildings, machinery, IT and stocks of vaccines for emergency or outbreaks.	More expenditure on healthcare and service could be associated with a higher capacity to treat cases[34,48,49].	2017	The World Bank<br>https://data.worldbank.org/indicator
	2. Infectious disease resilience index<br>A composite index that help identify countries that are potentially most vulnerable to poorly controlled infectious disease outbreaks because of a confluence of factors ranging across multiple domains, including political, economic, public health, medical, demographic, and disease dynamics. 0 indicating the country most vulnerable to infectious disease outbreaks and 1 indicating the most resilient country.	Countries with a high infectious disease resilience index are expected to be less vulnerable to an infectious disease outbreak.	Data published in 2016	Rand Corporation<br>https://www.rand.org/pubs/research_reports/RR1605.html
**Comorbidities**	1. DALY rates per 100,000 individuals from communicable, neonatal, maternal & nutritional diseases<br>Age-standardized DALY (Disability-Adjusted Life Year) rates per 100,000 individuals from communicable, neonatal, maternal & nutritional diseases. DALYs are used to measure total burden of disease – both from years of life lost and years lived with a disability. One DALY equals one lost year of healthy life.	Higher pre-pandemic prevalence of comorbidities including both communicable diseases (e.g. HIV) and non-communicable diseases [e.g. chronic obstructive pulmonary disease (COPD), hypertension coronary heart disease, diabetes, and obesity], have been found to increase COVID-19 mortality[16,22,34,50-52].<br><br>We included two specific comorbidities in our model – one communicable (HIV) and one non-communicable (diabetes). Other comorbidities including tuberculosis, COPD, hypertensive heart disease and obesity were included in sensitivity analyses.	2017	Our world in data<br>https://ourworldindata.org/burden-of-disease
	2. DALY rates per 100,000 individuals from non-communicable diseases<br>Age-standardized DALY rates per 100,000 individuals from non-communicable diseases (NCDs). DALYs are used to measure total burden of disease – both from years of life lost and years lived with a disability. One DALY equals one lost year of healthy life.		2017	
	3. Prevalence of HIV, total (% of population ages 15-49)<br>Prevalence of HIV refers to the percentage of people ages 15-49 who are infected with HIV.		2018, two countries (Seychelles, São Tomé and Príncipe) were missing.	The World Bank<br>https://data.worldbank.org/indicator
	4. Diabetes prevalence (% of population ages 20-79)<br>Diabetes prevalence refers to the percentage of people ages 20-79 who have type 1 or type 2 diabetes.		2019	

**Readiness**	1. COVID-19 test capacity<br>The ability to test for potential COVID-19 cases. There is no report case of COVID-19 in the WHO African Region when the data was released.	As a proxy for the capability to detect cases before the start of the pandemic, COVID-19 test capacity is expected to predict earlier detection of COVID-19 cases.	Based on newsletter released on 25 Feb. 2020	WHO Region office for Africa https://www.afro.who.int/health-topics/coronavirus-covid-19
	2. COVID-19 readiness status<br>Levels of readiness are assessed on the basis of information from an extensive survey that is given to WHO country offices in 47 countries in Africa. The survey questions are broadly grouped under nine response pillars: logistics; coordination; risk communication and community engagement; laboratory capacity; Points of Entry (PoE); surveillance; infection prevention and control; Rapid Response Teams (RRT); and case management.<br><br>Readiness status have been classified into three levels: limited, moderate and adequate.	As a proxy of the overall ability of local health care system to tackle the pandemic of COVID-19 before the start of the pandemic, countries with a more adequate COVID-19 readiness status likely predicts a lower mortality rate.	Based on newsletter released on 13 Feb. 2020; Three countries (Cape Verde, Mauritius, Seychelles) were missing.	
**Geography**	1. Number of borders<br>Number of countries with shared boundary.	As the indicator for imported cases from neighbouring countries, a higher number of borders predicts a high COVID-19 caseload and a higher mortality[36]. We use it here as a binary variable to distinguish island nations with presumed less porous borders.	2020	Data and Maps for ArcGIS (formerly Esri Data & Maps, https://www.arcgis.com/home/group.html?id=24838c2d95e14dd18c25e9bad55a7f82#overview)
	2. Latitude<br><br>The absolute degrees from equator.	Latitude is related to climate and may affect transmission rates[34,37,53].		
**COVID-19 testing**	1. Ratio of total COVID-19 mortality to reported COVID-19 mortality.<br><br>IHME used the excess mortality rate (observed all-cause mortality during the pandemic minus expected all-cause mortality based on pre-pandemic trends and seasonality) as a surrogate for the total COVID-19 mortality rate in all locations with sufficient data, and predicted the ratio of total COVID-19 mortality to reported COVID-19 mortality as a function of the infection-detection rate and location-specific fixed effects, using a Bayesian cascade model. Using the same model, ratios for all locations in the globe between March 2020 and May 2021were predicted.	Under-reporting and/or under-ascertainment of cases is widely anticipated to influence reported per capita mortality rates in Africa (as elsewhere) and could bias the analysis if varying between countries.	2021	IHME http://www.healthdata.org/special-analysis/estimation-excess-mortality-due-covid-19-and-scalars-reported-covid-19-deaths
	2. Days with testing policy index >= 2<br><br>3. Testing policy index on 1st November 2020	COVID-19 testing should reflect the level of detection of COVID-19 cases. Testing variables were used to correct the bias related	2020, four countries are missing (Equatorial Guinea, São Tomé and Príncipe, Guinea Bissau, Comoros)	OxCGRT https://www.bsg.ox.ac.uk/research/research-projects/coronavirus-

	The Oxford COVID-19 Government Response Tracker (OxCGRT) systematically collects publicly available information on several different indicators of response that governments have taken to respond to the COVID-19 pandemic, including school closures, travel restrictions etc. One of the indicators is testing policy, which record government policy on who has access to testing. The ordinal scores are shown below. The data is daily-based data and we calculated days with testing policy index above 2 between 25/02/2020 and 31/10/2020 as a predictor for per capita mortality in the first wave. Testing policy index at the start of the second wave on 1st November 2020 was also collected as a baseline predictor for per capita mortality in the second wave.<br><br>*0 – No testing policy*<br><br>*1 – Only those who both (a) have symptoms AND (b) meet specific criteria (e.g. key workers, admitted to hospital, came into contact with a known case, returned from overseas)*<br><br>*2 – testing of anyone showing COVID-19 symptoms*<br><br>*3 – open public testing (eg "drive through" testing available to asymptomatic people)*<br><br>*No data*	to the mortality such as under-reporting rate, data transparency, and testing coverage.		government-response-tracker
	4. Test data quality<br><br>The TIBA Pandemic Response Unit has created a new data set recording COVID-19 testing data as of 31 October 2020 for WHO Africa Region, and qualified the data quality into four categories.<br><br>*No data – viz. no data from official sources*<br><br>*Basic data – data that is published irregularly, or that does not distinguish between people tested and tests conducted*<br><br>*Satisfactory data – data that has none of the shortcomings of "basic data"*<br><br>*Good data – data that goes beyond daily numbers of tests or people tested. It may provide information on the positivity rate of tests, and on which tests were routine*		2020	GitLab https://git.ecdf.ed.ac.uk/epigroup/covid-19/tiba_testing_data/blob/master/TIBA_PRU_Testing_Data.xlsx

	surveillance or linked to contact tracing for example. It may distinguish in which laboratory tests have been conducted, or provide additional information on the gender or age or regional origin of people tested.			
	5. Total number of tests per 100K population as of 31 Dec. 2020<br><br>Africa CDC collected data on total number of tests per capita for each African country (https://africacdc.org/covid-19/). We obtained data as of 31 December 2020 from one paper published on Lancet on 24 March 2021. For nine countries (Algeria, Benin, Comoros, Eritrea, Liberia, Mauritius, Sao Tome and Principe, Seychelles, and Sierra Leone), the testing data were incomplete; For the United Republic of Tanzania, testing information was not reported for more than 2 weeks.		2020	Lancet https://www.thelancet.com/journals/lancet/article/PIIS0140-6736(21)00632-2/fulltext
**Interventions**	1. Area under the curve (AUC) of stringency index<br><br>2. Stringency index when cumulative deaths reached 0.1 per 10K population.<br><br>Stringency index represents policies on containment and closure, calculated from the data set for government mitigation responses to COVID-19 for WHO Africa Region, held by the TIBA Pandemic Response Unit. Stringency index is the average normalised strictness values of 12 subcategories of measures, excluding the governance and socio-economic measures and surveillance and testing from public health measures.	The stringency index reflects the government mitigation strategy in response to COVID-19. Policies including limits on gatherings, restrictions on international and national travel, and closing public services may be negatively associated with COVID-19 mortalities[22,37].	2020	GitLab https://git.ecdf.ed.ac.uk/epigroup/covid-19/tiba_pru_measures/blob/master/TIBA_PRU_measures.xlsx

Note. If no data were available for 2010 or later, the value was set as missing.

Supplementary Table 2. Hazard ratios and 95% confidence intervals for predictors for timing of the first case in univariable and multivariable cox regression model

Category	Variable	Univariable model	Multivariable model
**Demographics**	Population, total	2.002 (1.408-2.847)	
	Population density (people per sq. km of land area)	0.826 (0.608-1.122)	
	Urban population (% of total population)	1.265 (0.963-1.661)	1.404 (1.011-1.949)
	Population ages 65 and above (% of total population)	1.028 (0.754-1.402)	
	Sex ratio (Male/Female)	1.219 (0.900-1.652)	
**Socioeconomic**	GDP per capita (current US$)	1.198 (0.906-1.583)	
	Human development index	1.212 (0.904-1.626)	
**Travel**	Number of international airports	1.381 (1.007-1.892)	1.475 (1.017-2.139)
	Volume of international air travel	1.806 (1.358-2.403)	1.519 (1.095-2.106)
**Health care**	Current health expenditure (% of GDP)	0.603 (0.428-0.849)	0.754 (0.539-1.054)
	Infectious disease resilience index	1.253 (0.908-1.729)	
**Readiness**	COVID-19 test capacity, yes	3.643 (1.844-7.200)	3.861 (1.829-8.151)
	COVID-19 readiness status, adequate	2.628 (1.138-6.067)	
**Geography**	Number of borders, above 0	1.974 (0.817-4.773)	2.866 (1.122-7.316)
	Latitude	0.873 (0.646-1.179)	

Supplementary Table 3. Risk ratios and 95% confidence intervals of predictors for per capita mortality in the first wave in univariable and multivariable Poisson generalized linear mixed model

Category	Variable	Univariable model	Multivariable model (AICc=624.94)	Add day with testing policy index >= 2 (AICc=627.45)	Add testing data quality (AICc=627.46)	Add total number of tests per 100K population as of 31 Dec. 2020 (AICc=626.50)	Add AUC of stringency index (AICc=627.65)	Add stringency index when cumulative deaths reached 0.1 per 100K population (AICc=626.57)
**Demographics**	Population density (people per sq. km of land area)	0.751 (0.546-1.032)						
	Urban population (% of total population)	1.626 (1.221-2.167)	1.607 (1.252-2.063)	1.593 (1.238-2.049)	1.628 (1.261-2.102)	1.722 (1.306-2.271)	1.594 (1.218-2.086)	1.520 (1.163-1.986)
	Population ages 65 and above (% of total population)	1.192 (0.859-1.654)						
	Sex ratio (Male/Female)	1.171 (0.866-1.582)						
**Socioeconomic**	GDP per capita (current US$)	1.520 (1.054-2.192)						
	Human development index	1.552 (1.144-2.105)						
**Travel**	Number of international airports	1.135 (0.835-1.543)						
	Volume of international air travel	1.395 (1.050-1.855)	1.314 (1.040-1.660)	1.308 (1.035-1.653)	1.300 (1.024-1.649)	1.324 (1.050-1.668)	1310 (1.034-1.660)	1.268 (0.997-1.612)
**Health care**	Current health expenditure (% of GDP)	1.026 (0.752-1.399)						
	Infectious disease resilience index	1.653 (1.247-2.192)						
**Comorbidities**	DALY rates per 100,000 individuals from communicable, neonatal, maternal & nutritional diseases	0.760 (0.556-1.039)						
	DALY rates per 100,000 individuals from non-communicable diseases	0.908 (0.668-1.233)						
	Prevalence of HIV, total (% of population ages 15-49)	1.397 (1.044-1.870)	1.402 (1.104-1.780)	1.411 (1.110-1.793)	1.406 (1.107-1.784)	1.469 (1.144-1.886)	1.402 (1.104-1.780)	1.430 (1.127-1.816)
	Diabetes prevalence (% of population ages 20-79)	1.052 (0.766-1.444)						
**Readiness**	COVID-19 test capacity, yes	0.812 (0.424-1.557)						
	COVID-19 readiness status, adequate	1.239 (0.508-3.019)						
**Geography**	Number of borders, above 0	0.840 (0.288-2.456)						
	Latitude	1.467 (1.092-1.970)						
**COVID-19 testing**	Days with testing policy index >= 2, above median			1.128 (0.689-1.847)				
	Test data quality, satisfactory to good				1.128 (0.676-1.879)			
	Total number of tests per 100K population as of 31 Dec. 2020					0.846 (0.626-1.143)		
**Interventions**	AUC of stringency index						1.027 (0.743-1.420)	
	Stringency index when cumulative deaths reached 0.1 per 100K population							1.116 (0.877-1.549)

Supplementary Table 4. Odds ratios and 95% confidence intervals for outcome with respect to AUC of stringency index and mortality rate in multinomial logistic regression model.

Category	Variable	Univariable model			Multivariable model (AICc= 114.76)			Add days with testing policy index >= 2 (AICc= 119.17)			Add test data quality (AICc= 124.63)			Add total number of tests per 100K population as of 31 Dec. 2020 (AICc= 122.23)		
		High/Low	Low/High	High/High	High/Low	Low/High	High/High	High/Low	Low/High	High/High	High/Low	Low/High	High/High	High/Low	Low/High	High/High
**Demographics**	Population density (people per sq. km of land area)	0.912 (0.437-1.903)	0.578 (0.200-1.667)	0.136 (0.014-1.327)												
	Urban population (% of total population)	4.532 (1.208-16.993)	4.392 (1.176-16.403)	9.898 (2.348-41.731)	10.596 (1.441-77.928)	9.463 (1.276-70.204)	18.102 (2.260-144.997)	18.715 (2.187-160.166)	12.726 (1.587-102.050)	32.274 (3.483-299.082)	13.462 (1.627-111.404)	10.701 (1.307-87.625)	21.277 (2.401-188.595)	10.075 (1.461-69.487)	9.193 (1.295-65.249)	25.801 (3.090-215.412)
	Population ages 65 and above (% of total population)	0.345 (0.053-2.228)	0.778 (0.291-2.078)	1.004 (0.488-2.065)												
	Sex ratio (Male/Female)	1.619 (0.451-5.811)	0.941 (0.245-3.615)	2.280 (0.638-8.152)												
**Socioeconomic**	GDP per capita (current US$)	1.265 (0.307-5.212)	1.113 (0.254-4.882)	2.653 (0.768-9.167)												
	Human development index	2.349 (0.713-7.738)	2.746 (0.832-9.068)	4.233 (1.263-14.184)												
**Travel**	Number of international airports	1.702 (0.605-4.783)	1.226 (0.396-3.792)	1.685 (0.606-4.684)												
	Volume of international air travel	1.783 (0.294-10.815)	1.831 (0.305-10.990)	3.342 (0.613-18.228)												
**Health care**	Current health expenditure (% of GDP)	0.944 (0.424-2.103)	0.716 (0.297-1.728)	0.716 (0.304-1.687)												
	Infectious disease resilience index	2.202 (0.676-7.172)	3.666 (1.083-12.414)	5.866 (1.646-20.903)	4.508 (0.780-26.051)	8.015 (1.311-48.995)	10.136 (1.566-65.592)	6.969 (0.999-48.624)	11.907 (1.618-87.641)	15.274 (1.974-118.220)	4.151 (0.738-23.361)	7.447 (1.240-44.733)	9.388 (1.478-59.627)	4.165 (0.704-24.633)	7.941 (1.145-55.091)	16.323 (2.047-130.158)
**Comorbidities**	DALY rates per 100,000 individuals from communicable, neonatal, maternal & nutritional diseases	0.710 (0.283-1.779)	0.564 (0.218-1.463)	0.604 (0.240-1.518)												
	DALY rates per 100,000 individuals from non-communicable diseases	0.894 (0.376-2.127)	1.207 (0.534-2.727)	0.853 (0.362-2.005)												
	Prevalence of HIV, total (% of population ages 15-49)	1.198 (0.353-4.066)	2.127 (0.731-6.193)	1.777 (0.604-5.227)												
	Diabetes prevalence (% of population ages 20-79)	0.682 (0.268-1.739)	0.447 (0.126-1.590)	0.870 (0.418-1.809)												
**Readiness**	COVID-19 test capacity, yes	1.945 (0.322-11.756)	1.250 (0.221-7.084)	2.222 (0.375-13.179)												
**Geography**	Latitude	0.700 (0.279-1.758)	1.309 (0.552-3.105)	0.869 (0.366-2.065)												
**COVID-19 testing**	Days with testing policy index >= 2, above median							0.076 (0.006-0.965)	0.313 (0.029-3.403)	0.064 (0.004-0.971)						
	Test data quality, satisfactory to good										3.732 (0.348-40.057)	2.088 (0.200-21.836)	2.494 (0.205-30.406)			
	Total number of tests per 100K population as of 31 Dec. 2020													0.387 (0.032-4.674)	0.348 (0.026-4.761)	0.142 (0.009-2.211)

Note. COVID-19 readiness status and number of borders were excluded from these models because there is no country with adequate COVID-19 readiness status in the reference low/low level and no country with no borders in the high/high level.

Supplementary Table 5. Risk ratios and 95% confidence intervals of predictors for per capita mortality in the second wave in univariable Poisson generalized linear mixed model

Category	Variable	RR (95% CI)
**Demographics**	Population density (people per sq. km of land area)	0.779 (0.443-1.368)
	Urban population (% of total population)	1.109 (0.651-1.887)
	Population ages 65 and above (% of total population)	0.858 (0.473-1.554)
	Sex ratio (Male/Female)	0.739 (0.450-1.213)
**Socioeconomic**	GDP per capita (current US$]	1.367 (0.719-2.597)
	Human development index	1.945 (1.168-3.239)
**Health care**	Current health expenditure (% of GDP)	1.091 (0.654-1.823)
	Infectious disease resilience index	3.026 (1.999-4.582)
**Comorbidities**	DALY rates per 100,000 individuals from communicable, neonatal, maternal & nutritional diseases	0.584 (0.349-0.978)
	DALY rates per 100,000 individuals from non-communicable diseases	0.632 (0.386-1.034)
	Prevalence of HIV, total (% of population ages 15-49)	2.767 (1.864-4.109)
	Diabetes prevalence (% of population ages 20-79)	0.898 (0.516-1.561)
**Geography**	Number of neighbours, above 0	1.247 (0.204-7.609)
	Latitude	2.370 (1.520-3.695)
**First wave mortality**	Per 100K population mortality in the first wave	2.221 (1.435-3.438)
**First wave intervention**	AUC of stringency index in the first wave	1.098 (0.577-2.088)
**First wave testing**	Testing policy on November 1st, above 2	0.998 (0.353-2.822)
	Test data quality in the first wave, satisfactory to good	1.038 (0.360-2.990)
	Total number of tests per 100K population as of 31 Dec. 2020	1.322 (0.760-2.300)

Supplementary Table 6. Testing data in detail

Country	Source Quality	Type of Data	Data release frequency	Source
**Algeria**	Basic	N/A	Infrequently	http://www.aps.dz/sante-science-technologie/111985-coronavirus-320-nouveaux-cas-191-guerisons-et-10-deces
**Angola**	Basic	Samples analysed	Infrequently	OWiD, supplemented by https://www.minsa.gov.ao/TodasNoticias.aspx and https://governo.gov.ao/ao/noticias/taxa-de-positividade-e-de-6-6-por-cento/
**Benin**	Basic	Tests	Infrequently	https://www.gouv.bj/coronavirus/#mesures
**Botswana**	Basic	Tests	Infrequently	https://www.facebook.com/BotswanaGovernment/posts/3191898507559452
**Burkina Faso**	Satisfactory	Tests	Daily	OWiD, supplemented by https://www.sante.gov.bf/accueil
**Burundi**	Basic	Tests	Infrequently	http://minisante.bi/?p=735
**Cabo Verde**	Good		Daily	OWiD, supplemented by https://covid19.cv/conferencia-de-imprensa-sobre-a-covid-19-de-29-de-julho-de-2020/
**Cameroon**	Basic	Tests	Infrequently	https://twitter.com/drmanaouda
**Central African Republic**	Satisfactory	People tested	Daily	https://twitter.com/MSPCentrafrique?lang=en
**Chad**	Satisfactory	Samples analysed	Daily	https://www.facebook.com/ministeresantetchad/ and https://sante-tchad.org/communique-de-presse/
**Comoros**	No Data			
**Congo**	Basic	Tests	Infrequently	http://sante.gouv.cg/wp-content/uploads/2020/09/SITREP-N-100-COVID-19-CONGO-21-09-2020-1.pdf
**Côte d'Ivoire**	Good	Samples analysed	Daily	OWiD, supplemented by https://www.facebook.com/Mshpci
**Democratic Republic of the Congo**	Satisfactory	Samples analysed	Daily	OWiD, supplemented by https://riposte-epidemie-rdc.info/bulletins.php
**Equatorial Guinea**	Basic	Tests	Infrequently	https://www.guineaecuatorialpress.com/imgdb/2020/pyn9_INFORMEPERIODICO31072020Spanishfinal.pdf https://www.worldometers.info/coronavirus/country/equatorial-guinea/
**Eritrea**	No Data			
**Eswatini**	Good	Tests	Daily	http://www.gov.sz/index.php/covid-19-corona-virus/covid-19-press-statements-2020 and https://twitter.com/EswatiniGovern1
**Ethiopia**	Good	Tests	Daily	https://twitter.com/EPHIEthiopia
**Gabon**	Good	Tests	Daily	OWiD, supplemented by https://twitter.com/Covid19GOUVGA
**Gambia**	Good	Tests	Daily	http://www.moh.gov.gm/covid-19-report/
**Ghana**	Good	Samples analysed	Daily	https://www.ghanahealthservice.org/covid19/archive.php
**Guinea**	No Data			
**Guinea-Bissau**	Satisfactory	Tests	Daily	OWiD, supplemented by https://covid19gb.com/boletins/

**Kenya**	Good	Samples analysed	Daily	OWID, supplemented by https://twitter.com/MOH_Kenya
**Lesotho**	Basic	Samples analysed	Infrequently	OWID, supplemented by https://www.gov.ls/wp-content/uploads/2020/06/Update-on-Covid-19-status.pdf and https://www.gov.ls/official-statements-2/
**Liberia**	Basic	Samples analysed	Infrequently	http://moh.gov.lr/documents/reports/covid-19/2020/covid-19-sitrep-vol-17-2/
**Madagascar**	Satisfactory	Tests	Daily	http://www.sante.gov.mg/ministere-sante-publique/?s=COVID-19%3A+Situation
**Malawi**	Satisfactory	Tests	Daily	https://www.facebook.com/malawimoh
**Mali**	Good	Samples analysed	Daily	http://www.sante.gov.ml/index.php/actualites/communiques?start=84
**Mauritania**	Basic	Tests	Infrequently	http://www.sante.gov.mr/?lang=fr
**Mauritius**	No Data			
**Mozambique**	Satisfactory	Tests	Daily	http://www.misau.gov.mz/index.php/covid-19-boletins-diarios?limitstart=0
**Namibia**	Basic	Samples analysed	Infrequently	http://www.mhss.gov.na/home
**Niger**	Basic	People tested	Infrequently	https://reliefweb.int/report/niger/niger-coronavirus-covid-19-situation-report-08-8-21-june-2020
**Nigeria**	Satisfactory	Samples analysed	Daily	OWID, supplemented by https://ncdc.gov.ng/diseases/sitreps/?cat=14&name=An%20update%20of%20COVID-19%20outbreak%20in%20Nigeria
**Rwanda**	Satisfactory	Tests	Daily	https://www.rbc.gov.rw/index.php?id=717
**São Tomé and Príncipe**	Satisfactory	Tests	Daily	https://covid.ms.gov.st/st/
**Senegal**	Good	Tests	Daily	http://www.sante.gouv.sn/actualites
**Seychelles**	No Data			
**Sierra Leone**	Satisfactory	Tests	Daily	http://dhse.gov.sl/?s=SITREP
**South Africa**	Satisfactory	Tests	Daily	https://www.nicd.ac.za/diseases-a-z-index/covid-19/surveillance-reports/
**South Sudan**	No Data			
**Togo**	Good	People tested	Daily	OWiD, supplemented by: https://twitter.com/Covid19TG
**Uganda**	Satisfactory	Samples analysed	Daily	OWiD, supplemented by: https://twitter.com/MinofHealthUG
**United Republic of Tanzania**	No Data			
**Zambia**	No Data			
**Zimbabwe**	Good	Tests	Daily	http://www.mohcc.gov.zw/

OWiD, Our world in Data https://ourworldindata.org/coronavirus-testing

Supplementary Table 7. Sub-categories of measures in response to COVID-19 and their strictness scales

Category	Subcategory	Strictness Level	Score
**Governance and socio-economic measures**	Emergency administrative structures activated or established	None	0
		In place	1
**Lockdown**	Full lockdown	No lockdown	0
		Citizens required to stay at home nationwide	1
		Partial lockdown only in targeted areas of the country	2
	Partial lockdown	No partial lockdown	0
		Relaxed/Non-strict nationwide lockdown	1
		Full lockdown only in targeted areas of the country	2
**Movement restrictions**	Border closure	Borders open as normal	0
		Borders shut to some targeted countries, but not all affected countries	1
		Borders shut to all affected countries or a large list of countries	2
		Borders closed completely (except for repatriation, cargo and aid)	3
	Curfews	No specific curfew present	0
		Targeted curfew for some areas after 8pm	1
		Targeted curfew for some areas before 8pm	2
		Nationwide curfew after 8pm	3
		Nationwide curfew before 8pm	4
	Domestic travel restrictions	Able to travel freely throughout the whole country	0
		Very long-distance travel (e.g. domestic flights) suspended	1
		Long distance travel (e.g. between provinces) suspended	2

		Medium distance travel (outside your city) suspended	3
		Short distance travel (the next town/village) suspended	4
**Public health measures**	Isolation and quarantine policies	No policy	0
		Travellers from affected countries	1
		All travellers	2
		Any possible exposure	3
	Public health recommendations	None	0
		Recommendations in place	1
	Requirement to wear protective gear in public	No requirement	0
		Requirement for specific places only	1
		Requirement for most public places	2
	Surveillance and testing	No surveillance and monitoring	0
		Targeted/infrequent population surveillance	1
		Mass population surveillance	2
		Mass population testing	3
	Border health checks	No border health checks	0
		Basic test (e.g. temperature) for those from affected countries	1
		Basic (e.g. temperature) for all arrivals	2
		Advanced test for those from specific countries/Health document required from specific countries	3
		Advanced test for all arrivals/Health document required from all arrivals	4
**Social distancing**	Closure of businesses and public services	No closures	0
		Some businesses/services limited in hours	1
		Entertainment based businesses/services closed	2

		Entertainment based businesses/services closed, others limited in hours	3
		Entertainment and Hospitality closed	4
		Entertainment and Hospitality closed, others limited in hours	5
		All non-essential businesses closed	6
	Limit public gatherings	No limit on public gatherings	0
		Gatherings over 1000 suspended	1
		Gatherings over 100 suspended	2
		Non-religious gatherings over 50 suspended	3
		Gatherings over 50 suspended	4
		Non-religious gatherings over 10 suspended	5
		Gatherings over 10 suspended	6
		Restriction on gatherings less than 10	7
	Schools closure	All schools open	0
		Some levels of school closed	1
		All levels of school closed with exceptions for examinations	2
		All levels of school closed	3

Supplementary Table 8. Permission for the African shapefile used in the study

The African shapefile used in the study was obtained from Data and Maps for ArcGIS (formerly Esri Data & Maps, https://www.arcgis.com/home/group.html?id=24838c2d95e14dd18c25e9bad55a7f82#overview). Data and Maps for ArcGIS redistribution rights table (https://www.esri.com/content/dam/esrisites/en-us/media/legal/redistribution-rights/redist-rights-108.pdf) provides terms for internal and external use. The specific file we used–country.gdb–was under 'Yes 1'; detailed descriptions of the redistribution rights were shown below.

"Yes 1" Redistribution rights are granted by the data vendor for hard-copy renditions or static, electronic map images (e.g. .gif, .jpeg, etc.) that are plotted, printed, or publicly displayed with proper metadata and source/copyright attribution to the respective data vendor(s).
