# Supplementary material for: Predictors of COVID-19 epidemics in countries of the World Health Organization African Region
Source: Nat Med. 2021 Sep 3;27(11):2041–7. doi: 10.1038/s41591-021-01491-7 (PMC8604723; doi:10.1038/s41591-021-01491-7)
Supplement: Supplementary file 2 — Reporting Summary [file 41591_2021_1491_MOESM2_ESM.pdf]

nature research

Corresponding author(s): Feifei Zhang

Last updated by author(s): 30 July, 21

Reporting Summary

Nature Research wishes to improve the reproducibility of the work that we publish. This form provides structure for consistency and transparency in reporting. For further information on Nature Research policies, see our Editorial Policies and the Editorial Policy Checklist.

Statistics

For all statistical analyses, confirm that the following items are present in the figure legend, table legend, main text, or Methods section.

n/a	Confirmed	
☐	☒	The exact sample size (*n*) for each experimental group/condition, given as a discrete number and unit of measurement
☒	☐	A statement on whether measurements were taken from distinct samples or whether the same sample was measured repeatedly
☐	☒	The statistical test(s) used AND whether they are one- or two-sided<br>*Only common tests should be described solely by name; describe more complex techniques in the Methods section.*
☐	☒	A description of all covariates tested
☒	☐	A description of any assumptions or corrections, such as tests of normality and adjustment for multiple comparisons
☐	☒	A full description of the statistical parameters including central tendency (e.g. means) or other basic estimates (e.g. regression coefficient) AND variation (e.g. standard deviation) or associated estimates of uncertainty (e.g. confidence intervals)
☐	☒	For null hypothesis testing, the test statistic (e.g. *F*, *t*, *r*) with confidence intervals, effect sizes, degrees of freedom and *P* value noted<br>*Give P values as exact values whenever suitable.*
☒	☐	For Bayesian analysis, information on the choice of priors and Markov chain Monte Carlo settings
☒	☐	For hierarchical and complex designs, identification of the appropriate level for tests and full reporting of outcomes
☒	☐	Estimates of effect sizes (e.g. Cohen's *d*, Pearson's *r*), indicating how they were calculated

Our web collection on statistics for biologists contains articles on many of the points above.

Software and code

Policy information about availability of computer code

Data collection	R version 3.6.3 (R Foundation for Statistical Computing, Vienna, Austria, 2020)
Data analysis	The R version 3.6.3 (R Foundation for Statistical Computing, Vienna, Austria, 2020) was used in all statistical analyses. R packages used for model fitting included survival, lme4, nnet, and mgcv. All data are made available via figshare at https://doi.org/10.6084/m9.figshare.15022503.

For manuscripts utilizing custom algorithms or software that are central to the research but not yet described in published literature, software must be made available to editors and reviewers. We strongly encourage code deposition in a community repository (e.g. GitHub). See the Nature Research guidelines for submitting code & software for further information.

Data

Policy information about availability of data

All manuscripts must include a data availability statement. This statement should provide the following information, where applicable:

- Accession codes, unique identifiers, or web links for publicly available datasets
- A list of figures that have associated raw data
- A description of any restrictions on data availability

Data on COVID-19 cases and deaths were from the WHO COVID-19 Dashboard (https://covid19.who.int/info/). Data source for predictors included the World Bank, United Nations, Rand Corporation, Our world in data, WHO Region office for Africa, Data and Maps for ArcGIS (formerly Esri Data & Maps), Institute for Health Metrics and Evaluation, the Oxford COVID-19 Government Response Tracker, and Africa CDC (links are provided in Supplementary table 1). All data are made available via figshare at https://doi.org/10.6084/m9.figshare.15022503.

Field-specific reporting

Please select the one below that is the best fit for your research. If you are not sure, read the appropriate sections before making your selection.

☒ Life sciences ☐ Behavioural & social sciences ☐ Ecological, evolutionary & environmental sciences

For a reference copy of the document with all sections, see nature.com/documents/nr-reporting-summary-flat.pdf

Life sciences study design

All studies must disclose on these points even when the disclosure is negative.

Sample size	We focus on the variations of COVID-19 epidemics within the WHO African Region and therefore the sample is the 47 Member States within the region. The sample is sufficient according to the one predictor in five to nine events rule (van Smeden M et al 2016, Vittinghoff E et al 2007), because we have included a maximum of 6 predictors in all our multivariable models.
Data exclusions	All 47 Member States were included in the first case analyses. However, Tanzania discontinued reporting of COVID-19-related deaths from 8 May 2020, and Burundi, Eritrea and Seychelles were outliers (0.009, 0 and 0 per 100K population first wave mortality rates, respectively). São Tomé & Príncipe, as well as Seychelles, had missing data on the prevalence of HIV. All five countries were therefore excluded from the mortality rate analyses, giving a sample size of 42.
Replication	All codes were re-run by a second author and all attempts at replication were successful.
Randomization	Randomization is not relevant to the study because it is an observational study.
Blinding	Blinding is not relevant to the study because it is an observational study.

Reporting for specific materials, systems and methods

We require information from authors about some types of materials, experimental systems and methods used in many studies. Here, indicate whether each material, system or method listed is relevant to your study. If you are not sure if a list item applies to your research, read the appropriate section before selecting a response.

Materials & experimental systems

n/a	Involved in the study	
☒	☐	Antibodies
☒	☐	Eukaryotic cell lines
☒	☐	Palaeontology and archaeology
☒	☐	Animals and other organisms
☒	☐	Human research participants
☒	☐	Clinical data
☒	☐	Dual use research of concern

Methods

n/a	Involved in the study	
☒	☐	ChIP-seq
☒	☐	Flow cytometry
☒	☐	MRI-based neuroimaging
